# Key Role of Alphaproteobacteria and Cyanobacteria in the Formation of Stromatolites of Lake Dziani Dzaha (Mayotte, Western Indian Ocean)

**DOI:** 10.3389/fmicb.2018.00796

**Published:** 2018-05-22

**Authors:** Emmanuelle Gérard, Siham De Goeyse, Mylène Hugoni, Hélène Agogué, Laurent Richard, Vincent Milesi, François Guyot, Léna Lecourt, Stephan Borensztajn, Marie-Béatrice Joseph, Thomas Leclerc, Gérard Sarazin, Didier Jézéquel, Christophe Leboulanger, Magali Ader

**Affiliations:** ^1^UMR CNRS 7154 Institut de Physique du Globe de Paris, Sorbonne Paris Cité, Université Paris Diderot, Centre National de la Recherche Scientifique, Paris, France; ^2^Université Lyon 1, UMR CNRS 5557 / INRA 1418, Ecologie Microbienne, Villeurbanne, France; ^3^UMR 7266 CNRS-Université de la Rochelle, LIttoral ENvironnement Et Sociétés, La Rochelle, France; ^4^School of Mining and Geosciences, Nazarbayev University, Astana, Kazakhstan; ^5^Museum National d’Histoire Naturelle, Institut de Minéralogie, de Physique des Matériaux et de Cosmochimie, UMR 7590 CNRS Sorbonne Universités, Université Pierre et Marie Curie, Institut de Recherche pour le Développement UMR 206, Paris, France; ^6^UMR MARBEC, IRD, Ifremer, CNRS, Université de Montpellier, Sète, France

**Keywords:** stromatolites, alkaline lake, Pleurocapsales, Mg-silicate, Alphaproteobacteria, anoxygenic phototrophic bacteria, aragonite, hydromagnesite

## Abstract

Lake Dziani Dzaha is a thalassohaline tropical crater lake located on the “Petite Terre” Island of Mayotte (Comoros archipelago, Western Indian Ocean). Stromatolites are actively growing in the shallow waters of the lake shores. These stromatolites are mainly composed of aragonite with lesser proportions of hydromagnesite, calcite, dolomite, and phyllosilicates. They are morphologically and texturally diverse ranging from tabular covered by a cauliflower-like crust to columnar ones with a smooth surface. High-throughput sequencing of bacterial and archaeal 16S rRNA genes combined with confocal laser scanning microscopy (CLSM) analysis revealed that the microbial composition of the mats associated with the stromatolites was clearly distinct from that of the *Arthrospira*-dominated lake water. Unicellular-colonial Cyanobacteria belonging to the *Xenococcus* genus of the Pleurocapsales order were detected in the cauliflower crust mats, whereas filamentous Cyanobacteria belonging to the *Leptolyngbya* genus were found in the smooth surface mats. Observations using CLSM, scanning electron microscopy (SEM) and Raman spectroscopy indicated that the cauliflower texture consists of laminations of aragonite, magnesium-silicate phase and hydromagnesite. The associated microbial mat, as confirmed by laser microdissection and whole-genome amplification (WGA), is composed of Pleurocapsales coated by abundant filamentous and coccoid Alphaproteobacteria. These phototrophic Alphaproteobacteria promote the precipitation of aragonite in which they become incrusted. In contrast, the Pleurocapsales are not calcifying but instead accumulate silicon and magnesium in their sheaths, which may be responsible for the formation of the Mg-silicate phase found in the cauliflower crust. We therefore propose that Pleurocapsales and Alphaproteobacteria are involved in the formation of two distinct mineral phases present in the cauliflower texture: Mg-silicate and aragonite, respectively. These results point out the role of phototrophic Alphaproteobacteria in the formation of stromatolites, which may open new perspective for the analysis of the fossil record.

## Introduction

Microbialites are sedimentary structures which construction is microbially mediated, and may show stromatolitic (laminated) or thrombolytic (clotted) fabrics (Burne and Moore, 1987). Fossil microbialites, and in particular fossil stromatolites, can be traced back to 3700 Myr ago (Nutman et al., 2016) and are thus considered among the oldest fossil records of life on Earth (Allwood et al., 2006; Nutman et al., 2016). They were particularly abundant during the Proterozoic Eon, especially around 1250 Myr ago (Riding, 2006; Peters et al., 2017). By analogy with modern stromatolites developing today, it has been proposed that fossil stromatolites were formed by microbial mats dominated by Cyanobacteria (Altermann et al., 2006; Bosak et al., 2009). However, Precambrian and Cambrian stromatolites rarely contain observable fossils of microorganisms. Presumed microfossils constituted of organic globules associated with aragonite were found in 2724-Myr-old stromatolites from the Tumbiana Formation (Fortescue Group, Australia) (Lepot et al., 2008). The oldest potential filamentous carbonate-encrusted cells identified in the Transvaal Supergroup in South Africa have been dated at 2,500–2,300 Myr (Klein et al., 1987). However, the earliest undisputed occurrence of fossil filamentous Cyanobacteria (*Girvanella* genus) is much less ancient, dated at 750–700 Myr (Riding, 1982). The lack of microfossils in ancient stromatolites has resulted in the proposition of other hypotheses for their formation, including formation by anoxygenic photosynthesis (Bosak et al., 2007) or purely abiotic processes (Lowe, 1994; Grotzinger and Rothman, 1996; McLoughlin et al., 2008). Although environmental conditions undoubtedly changed through geological time, detailed biological and mineralogical studies of modern microbialites developing in marine or lacustrine environments offer a unique opportunity to obtain new information which will help in the interpretation of the fossil record.

Cyanobacteria play a key role in the formation of microbialites, being usually the main primary producers in the microbial mats associated with modern stromatolites. They have been shown to influence the shape of growing stromatolites in different ways. First, filamentous Cyanobacteria can trap and bind sedimentary particles and depending on the length of filaments, the size of the trapped sedimentary particles is variable (Franz et al., 2015). Consequently, it has been postulated that the grain size distributions observed in fossil stromatolites were determined by the size of Cyanobacteria or other filamentous microorganisms (Franz et al., 2015). Second, the conical shape of certain modern stromatolites has indeed been attributed to thin filamentous Cyanobacteria forming small cones in response to light, even in the absence of lithification (Walter et al., 1976; Bosak et al., 2009). Finally, the oxygen produced by Cyanobacteria at the tip of lithifying cones induces the formation of contorted laminations, in which oxygen bubbles can be trapped (Bosak et al., 2009). Such contorted laminations with supposed fossil oxygen bubbles were traced back in 2700-Myr-old stromatolites from the Meentheena Member of the Tumbiana Formation in Australia but not in older formations (Bosak et al., 2009).

Furthermore, the metabolism of Cyanobacteria can favor the formation of carbonate minerals. Photosynthetic activity is indeed an alkalinizing process that may promote carbonate precipitation in the vicinity of Cyanobacteria (Dupraz and Visscher, 2005; Visscher and Stolz, 2005; Dupraz et al., 2009). During photosynthesis, Cyanobacteria assimilate CO_2_ mainly through the active import of bicarbonate ions and less through the passive diffusion of CO_2_ into their cells (Jansson and Northen, 2010). Within the cell, carbonic anhydrase catalyzes the conversion of bicarbonate ions to CO_2_ and produces OH^-^. It is hypothesized that both CO_2_ uptake and OH^-^ release may induce a rise of pH and an increase of the activity of the CO_3_^2-^ ion and therefore the possible precipitation of Mg or Ca carbonate depending on cation availability (Merz, 1992; Riding, 2006; Jansson and Northen, 2010). The nature of the precipitating carbonate can be influenced by some Cyanobacteria as those belonging to the Pleurocapsales and Chroococcales orders. In the microbialites from the hyperalkaline Lake Alchichica in Mexico, Pleurocapsales induce the formation of aragonite, in which they become encrusted, at the expense of hydromagnesite which is the major component of the microbialites (Couradeau et al., 2013; Gerard et al., 2013; Saghai et al., 2015). This may be due to the properties of the sheaths of the Pleurocapsales in which cations can be accumulated.

The mechanism of lamination formation in growing stromatolites is still an open question and seems to involve different species of Cyanobacteria and heterotrophic bacteria. A model for the formation of biologically induced laminations was proposed for the Bahamian stromatolites. It involves the temporal succession of three different microbial mats dominated by filamentous Cyanobacteria trapping and binding carbonate sand grains, subsequently by heterotrophic bacteria forming thin crusts of microcrystalline aragonite, and finally by coccoid endolithic Cyanobacteria favoring cementation of the grains (Reid et al., 2000). It has been shown that heterotrophic degradation of extracellular polymeric substances (EPS) by sulfate reducing bacteria (SRB) was a major factor controlling mineral precipitation and micritization in microbial mats associated with stromatolites [for reviews (Decho, 2010; Dupraz and Visscher, 2005)]. Recently, it has been observed that the highest degrees of lamination were related to the presence of coccoid Cyanobacteria and pervasive aragonite precipitation in the microbial mats rather than to the presence of filamentous Cyanobacteria trapping and binding carbonate sand grains (Suosaari et al., 2016).

Comparatively to Cyanobacteria, less attention has been paid to the calcifying role of the phototrophic purple bacteria which are abundant in stromatolitic mats (e.g., Papineau et al., 2005; Saghai et al., 2015). Yet, the Earth atmosphere and ocean were probably largely anoxic before 2,500 Myr and anoxygenic phototrophy most likely predated oxygenic photosynthesis (Blankenship, 2010; Xiong et al., 2000). Phototrophic bacteria may thus have been important stromatolite builders prior to the appearance of oxygenic photosynthesis. It that sense, it has been shown experimentally that both anoxygenic photosynthetic and photoheterotrophic purple bacteria are able to promote carbonate precipitation (Bosak et al., 2007; Bundeleva et al., 2012). However, experimental evidence suggests also that they should be far less efficient in promoting carbonate precipitation than oxygenic photosynthetic Cyanobacteria in natural environments (Bundeleva et al., 2012). The potential role of phototrophic bacteria in the formation of stromatolites remains thus unclear and, so far, evidences based on the study of modern microbialites indicating that their formation may occur through anoxygenic photosynthesis are still lacking.

We describe for the first time the microbialites developing in Lake Dziani Dzaha, a tropical crater lake located on Mayotte Island (Comoros archipelago, Western Indian Ocean). Dziani Dzaha is a shallow and turbid lake located in close proximity to the ocean (Leboulanger et al., 2017). The lake water has a salinity 1.5 times that of sea water, a high level of dissolved organic matter (average DOC = 7186 ± 843 μmol L^-1^) and an extremely high alkalinity (100 times seawater) (Leboulanger et al., 2017). The green color of the lake is due to a permanent bloom of filamentous Cyanobacteria belonging to the *Arthrospira* genus (Leboulanger et al., 2017). Stromatolites with variable degrees of laminations and different surface morphologies are developing on the shores of the lake. Most of the dome-shaped or small columnar stromatolites are covered by a hard cauliflower-like crust, whereas the large columnar stromatolites are characterized by friable, smooth to granular surfaces. The purpose of the present study was: (i) to determine if the different types of stromatolites were associated with different types of microbial mats, (ii) to identify particular microbial lineages responsible for the building of stromatolites by trapping, binding or precipitating minerals, (iii) to discriminate among the mineral phases of the stromatolites those for which the formation may have been bioinduced. Toward this aim, the microbial diversity of the different mats was analyzed using Illumina high-throughput 16S rRNA gene amplicon sequencing. The bulk mineralogy of the stromatolites was determined using X-ray powder diffraction and the geochemistry of the lake water was interpreted in terms of mineral-solution equilibria. Detailed observations using confocal laser scanning microscopy (CLSM), Raman spectroscopy, scanning electron microscopy (SEM), and laser microdissection permitted us to explore the relations between the formation of mineral phases and the different microbial lineages at the microscale.

## Materials and Methods

### Sample Collection and Fixation

**FIGURE 1:**
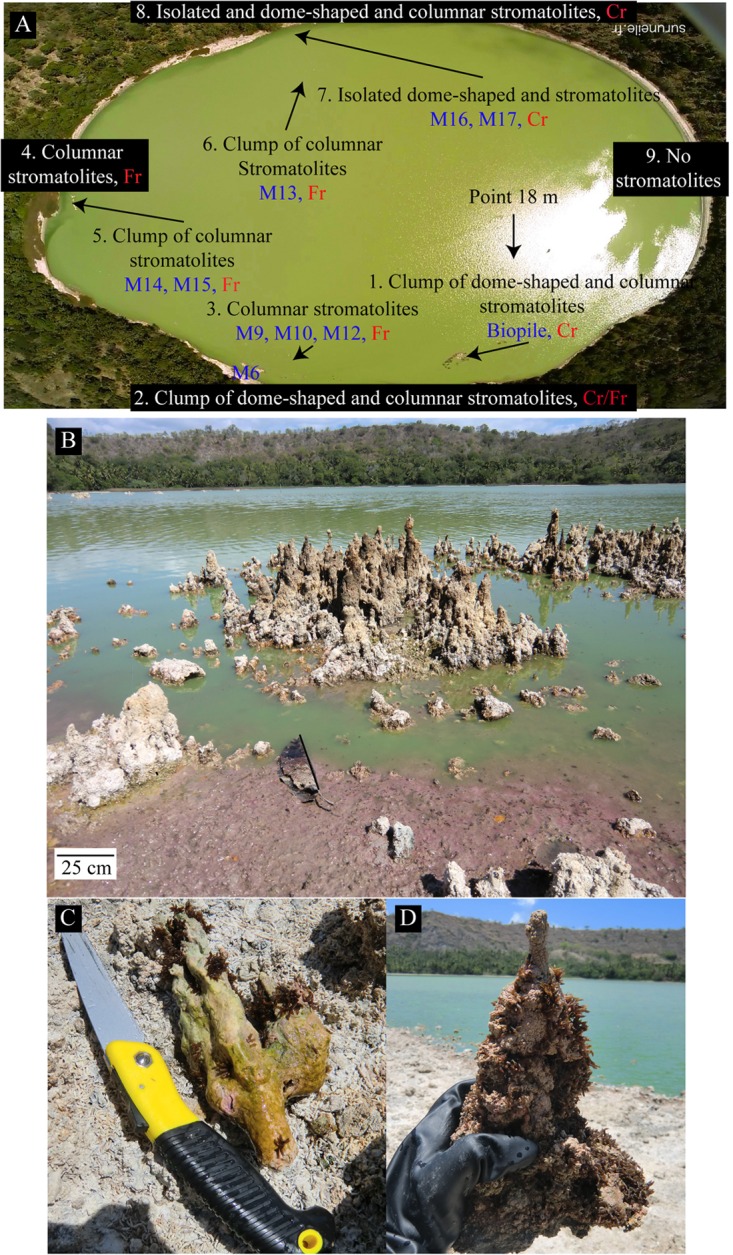
**(A)** Aerial view of the Dziani Dzaha Lake in October 2014 showing the positions of the sampled stromatolites (M6–M19) and the points where water was collected (point 18 m) for the chemical analysis. A general description of the types of the different stromatolites found on the North (8), South (2), West (4), and East (9) shores is indicated in the red boxes. No stromatolites or only scarce friable flat crusts were found on the East shore. Cr and Fr describe the surface of stromatolites, Cr: crust, Fr: friable. **(B)** Global view of the lake with clumps of emerged columnar stromatolites on the south part of the lake. The green color of the water is due to the high abundance of Cyanobacteria. The purple color visible on the shore may be related to purple bacteria. **(C)** A columnar stromatolite (M6) collected 30 cm under the water surface covered by a greenish to purple microbial mat. **(D)** A columnar stromatolite (M16), collected 20 cm under the water surface with a cauliflower-like texture covered by a purple to green thin biofilm. Note that both stromatolites are partly covered by fly pupae except the top few centimeters of the M16 stromatolite that was probably formed after the last fly laying.

Lake Dziani Dzaha is located on the island of “Petite Terre” (12°46′15.6″S; 45° 17′19.2″E), belonging to the island complex of Mayotte (Leboulanger et al., 2017). The field campaign of October 2014 was conducted at the end of the dry season, when the lake water was at its lowest level. The water column was not stratified, except for dissolved oxygen which decreased with depth (55.5, 0.3, and 0 μM at surface, 1 and 2.5 m below the surface, respectively). A number of stromatolites were emerged from the lake, while others were still immerged. All stromatolite samples presented in this study were taken from the oxic zone between 0 and 1 m water depth in October 2014. The annual drawdown of the lake is about 70 cm, the highest water level being reached during the rainy season from December to March. Consequently, the stromatolites sampled above 30 cm depth in October 2014 were probably located at the transition between the oxic and the anoxic zones when the lake was at its highest level. The dissolved oxygen concentrations recorded in April 2014 were 405, 88.8, and 0 μM, at surface, 1 and 2.5 m below the surface, respectively. Stromatolites were sampled all around the lake (**Figure 1** and Supplementary Table S1). On the west shore, only scarce flat crusts were found and were not analyzed in this study.

Three stromatolites covered by a cauliflower crust (one flat and two small columnar of about 20 cm high) and the top of three columnar stromatolites of about 1 m high without cauliflower crust (two granular and one smooth) were chosen (**Figure 1**, Supplementary Figures S2, S3, and Supplementary Table S1) and prepared in three different ways for CLSM. Samples were fixed immediately on the field in RNAlater^®^ (Thermo Fisher Scientific, Waltham, MA, United States), 50% ethanol in phosphate buffered saline or formalin neutral buffered 10% (Merck KGa, Darmstadt, Germany) solutions and preserved on ice (for a maximum period of 2 h) before being transferred in a freezer at -20°C. Samples for DNA extractions were not fixed; they were kept at 4°C (for a maximum period of 2 h) and transferred at -20°C as soon as possible. Samples were fixed in RNAlater^®^ to preserve the pigmentation of Cyanobacteria and Purple Bacteria. Otherwise, samples were fixed in formaldehyde 4% and ethanol 50%, and then embedded in LR-white resin (Polysciences, Warrington, PA, United States) to analyze correlations between microorganisms and mineral phases by concomitant CLSM/Raman and SEM analysis as previously described in Couradeau et al. (2013), Gerard et al. (2013). Formaldehyde/ethanol fixed samples were dried by the CO_2_ critical-point method using Emcpd300 (Leica Microsystems GmbH, Wetzlar, Germany) at Institut de Biologie Paris-Seine (IBPS, Paris, France) for SEM analysis. Finally, unfixed stromatolites were also included in epoxy resin for SEM analysis.

### X-Ray Powder Diffraction (XRPD)

The bulk mineralogy of microbialites was determined by XRPD. About 1 g of each sample was ground in an agate mortar. The powder was deposited on a single crystal silicon sample holder. Measurements were performed with an Empyrean Panalytical diffractometer using Cu-Kα radiation. Data were recorded between 4 and 90° (2 θ) with a step of 0.013° and a total counting time of half an hour. The PANalytical Highscore Plus software (Degen et al., 2014) was used for background subtraction, peak identification and matching with XRD patterns of reference compounds from the International Crystal Structure Database (ICSD, FIZ Karlsruhe, Germany; US Institute of Standards and Technology, United States).

### Physicochemical Parameters of the Lake Dziani Dzaha Water

The following physicochemical parameters of the lake water have been measured during five different campaigns (2012–2015): pH, temperature, concentrations of dissolved O_2_, major anionic and cationic species, conductivity and alkalinity. All the methods used are described in Leboulanger et al. (2017). Vertical profiles were taken using either a MPP350 probe connected to a Multi 350i data logger (WTW GmbH) or an YSI 600XLM probe (YSI) to measure pH, dissolved O_2_, temperature and conductivity. The total alkalinity was determined by titration with 0.01 M HCl after 100 times dilution. Major cations were analyzed to ±5% by inductively coupled plasma atomic emission spectroscopy (ICP-AES iCAP6200 Thermo Fisher). Sulfate was measured to ±3% using 20-fold diluted samples by ionic chromatography (ICS 1100 Thermo Fisher). Chloride was titrated using a standard AgNO_3_ solution (0.5 M), after acidification with analytical grade HNO_3_ (to prevent Ag_2_CO_3_ precipitation). Dissolved silicate and soluble reactive phosphorus were measured using classical methods of spectrometry developed for seawater analysis (Amino and Kérouel, 2004). Dissolved organic carbon (DOC) was measured using a TOC analyzer (Shimadzu, TOC V CHS/CHN) after acidification with 1% H_3_PO_4_ and 1/10 dilution. The concentration of aqueous hydrogen sulfide [ΣH_2_Saq = H_2_Saq + HS^-^ + S_2_^-^ (trace) + RS(-II) ion complexes, clusters, nanoparticles, and colloids] was determined by a colorimetric method described in Reese et al. (2011). In this method, hydrogen sulfide reacts with N,N′-dimethyl-1,4-phenylene-diamine in H_2_N-SO_3_H (at 120 g/l). Leucomethylene blue complex is formed and oxidized by ferric ions to methylene blue having a maximum absorbance at 664 nm.

### Thermodynamic Analysis

The saturation state of the surface waters with respect to selected mineral phases was evaluated from the relation:

$$\Omega =\log\frac{Q}{K}$$

where Ω is the saturation index, *Q* is the reaction quotient or ion activity product, and *K* is the equilibrium constant for the reaction of dissolution of the mineral under consideration. The reaction quotient is defined by:

$$Q=\underset{i}{\Pi }a_{i}^{n_{i}}$$

where *a*_i_ is the activity of the subscripted aqueous species and *n*_i_ is the stoichiometric coefficient of that species in the reaction. Values of *a*_i_ were computed with the PHREEQC computer program (Parkhurst and Appelo, 1999) using an extended Debye–Hückel model for the activity coefficients. Mineral stability diagrams were constructed using equilibrium constants calculated with the SUPCRT92 program (Johnson et al., 1992).

### DNA Extraction and High-Throughput Sequencing

DNA extractions were performed using the Powersoil DNA kit (Mo Bio, Carlsbad, CA, United States). The biofilms associated with the stromatolites were discriminated according to their color (green or red) using a sterile scalpel only when this was possible. For the stromatolites M10, M12, and M15 a distinct green or red area of a few centimeters was distinguished (Supplementary Figures S3D,G). The mats were dissected in accordance to the colors observed (M10, M12, and M15). It was not possible to separate the different colors from the cauliflower crust for DNA extraction because the green and purple/pink colors were overlapping (M16 and M17).

Amplification of the V3–V5 region of the 16S rRNA genes was performed for both Bacteria and Archaea, using the primers 357F (Schuurman et al., 2004) and 926R (Walters et al., 2016) and 519F and 915R (Hugoni et al., 2015), respectively. High-throughput sequencing was achieved after a multiplexing step, using a HiSeq Rapid Run 300bp PE technology on an Illumina HiSeq 2500 system (GATC Biotech, Konstanz, Germany).

### Bioinformatic Analysis and Sequence Processing

Bacterial and archaeal 16S rRNA paired-end reads were merged with a maximum of 10% mismatches in the overlap region using FLASH (Magoc and Salzberg, 2011). Denoising procedures consisted in discarding reads with no expected length (i.e., expected size between 450 and 580 bp, 370 and 580 bp, for bacterial and archaeal 16S rRNA genes, respectively) and the ones containing ambiguous bases (N). After dereplication, the clusterisation tool ran with SWARM (Mahe et al., 2014). In the present work, aggregation distance was equal to 3. Chimeras were then removed using VSEARCH (Rognes et al., 2016) and low abundance sequences were filtered at 0.005% [i.e., to keep OTUs accounting for at least 0.005% of all sequences (Bokulich et al., 2013), in order to remove singletons from the datasets]. Taxonomic affiliation was performed with both RDP Classifier (Wang et al., 2007) and Blastn+ (Camacho et al., 2009) against the 128 SILVA database (Pruesse et al., 2007). This procedure was automated in the FROGS pipeline (Escudie et al., 2017).

To compare samples, a normalization procedure was applied to randomly resample down to 51,730 and 21,673 sequences for bacterial and archaeal 16S rRNA genes, respectively.

Differences in bacterial community structure were visualized using non-metric multidimensional scaling (NMDS) ordinations on abundance-based (Bray–Curtis) dissimilarity matrices. Using non-parametric permutation-based multivariate analysis of variance (PERMANOVA, function adonis in R package vegan, (Anderson, 2001) on abundance-based (Bray–Curtis) dissimilarity matrices, we tested for significant differences in community structure. These analyses were performed with the VEGAN package^1^ in R.

The Illumina sequence data generated in this study were deposited here: http://www.ebi.ac.uk/ena/data/view/PRJEB25249. The GenBank accession numbers for the Sanger sequence data are MH036348 for T12-5C and MH036349 for M17-60.

### Scanning Electron Microscopy (SEM)

Samples for SEM were divided into three groups: the first group was dried using the CO_2_ critical-point method; the second embedded in LR-white resin and cut in 1–2 mm thick slices, and the third embedded in epoxy, cut and mounted on microscopy slides, and all samples were coated with a 15 nm carbon layer.

The SEM was performed at the Service Commun de Microscopie Electronique à Balayage (UPMC, Paris, France) using a Zeiss SUPRA 55 VP Field Emission Scanning Electron Microscope. Images were collected using secondary electron detectors (Everhart-Thornley for high voltage mode, VPSE for variable pressure mode and InLens for low voltage mode) and a backscattered electron detector (AsB). Accelerating voltage ranged from 3 to 15 kV at variable pressures and high current (up to 1 nA) or was fixed at 3 kV under high vacuum and low current (down to 10 pA). Alternatively, a Zeiss EVO MA 10 microscope equipped with both a back-scattered electron (BSE) detector and a secondary electron (SE2) detector, as well as a Zeiss Auriga FEG-FIB microscope were used (Plateforme de Microscopie Electronique, IPGP, Paris, France). Elemental microanalyses and mapping were performed using Energy Dispersive X-ray (EDX) spectrometers (PGT Sahara and Bruker Quanta 200).

### Combined CLSM and Raman Spectrometry Imaging

Confocal laser scanning microscopy and Raman analyses were performed as previously described in Gerard et al. (2013) using an Olympus FluoView FV1000 confocal microscope (Olympus Tokyo, Japan) combined with an Invia Raman Spectrometer (Renishaw, Wotton-under-Edge, United Kingdom). Samples fixed with RNAlater^®^ or formaldehyde, unstained or stained were observed using an oil immersion objective (Olympus UPSLAPO X60). DNA staining of microorganisms were performed either with DAPI (4′,6-diamidino-2-phenylindole) at 1 to 10 μg.ml^-1^ or Syto9^®^ Green Fluorescent Nucleic Acid Stain at 10 μM (Thermo Fisher Scientific, Waltham, MA, United States) for 10 min at room temperature in the dark, followed by two washings with sterile Milli-Q water. For concomitant CSLM/Raman image acquisitions on samples embedded in LR-white resin, a water immersion LUMPLFL 60×W objective (Olympus; 60× magnification) was used. Fluorescence image stacks were obtained with concomitant excitation at wavelengths of 405, 488, and 543 nm by collecting the emitted fluorescence between 425–475, 500–530, and 560–660 nm, respectively. For Raman analyses we used a 785 nm laser source and proceeded as described in Gerard et al. (2013). Briefly, dynamic line–scanning Raman mapping (Renishaw Streamline) was performed in the range 387–1538 cm^-1^ by scanning the sample over selected areas (20 s pt^-1^) by using a motorized PRIORTM stage. Laser intensity was set at 300 mW. Light was 1200 grooves per millimeter and the signal was analyzed with a RECAM charge-coupled device detector. Compositional maps representing the intensity distributions of characteristic peaks were determined using the software Wire 3.2.

### Laser Microdissection, Whole Genome Amplification (WGA) and Cloning-Sequencing of 16S RNA Genes

Small individual Pleurocapsales colonies and associated coccoid and filamentous cells were isolated using a Zeiss PALM MicroBeam apparatus installed in a clean room. We then used the REPLI-g Single Cell Kit (Qiagen, Hilden, Germany) to amplify whole genomic DNA of the microdissected cells. Bacterial 16S rRNA genes were then amplified by PCR using the bacterial specific primer 27F (5′-AGAGTTTGATCCTGGCTCAG-3′) and with the universal prokaryotic reverse primer 1492R (5′-GGTTACCTTGTTACGACTT-3′). One microliter of 1/10 diluted amplified genomic DNA was used in a reaction buffer volume of 25 μL containing dNTPs (10 nmol each), 20 pmol of each primer and 1 U of GoTaq polymerase (Promega, France). PCR was performed under the following conditions: 35 cycles (denaturation at 94°C for 15 s, annealing at 55°C for 30 s, extension at 72°C for 2 min) preceded by 2 min denaturation at 94°C, and followed by 7 min extension at 72°C. Cloning was done using the Topo TA Cloning system (Thermo Fisher Scientific, Waltham, MA, United States) following the instructions provided by the manufacturer. After plating, positive transformants were screened by PCR amplification of inserts using flanking vector primers and the PCR products were partially sequenced (≈700 bp) by GATC Biotech (Konstanz, Germany) using flanking vector primer T7 (5′-TAATACGACTCACTATAGGG-3′). At least one representative clone per phylotype or Operational Taxonomic Unit (OTU, group of sequences sharing >97% identity) was fully sequenced using flanking vector primer M13R (5′- CAGGAAACAGCTATGAC -3′) for detailed phylogenetic analysis.

### Phylogenetic Analyses

Taxonomic affiliations at the phylum level were first obtained by comparing several portions of partial 16S rRNA gene sequences with sequences of the GenBank database using BLAST [Basic Local Alignment Search Tool (Altschul et al., 1997)]. Representative clones of the dominant phyla were then fully sequenced and analyzed with the ARB software (Ludwig et al., 2004) by using the 123 SILVA database (Pruesse et al., 2007; Quast et al., 2013; Yilmaz et al., 2014). The sequences were first aligned with the SINA online aligner (Pruesse et al., 2012) and then added in the ARB guide tree using the ARB parsimony tool. The phylogenetic tree was constructed by adding to the aligned sequences, sequences of the closest cultivated bacteria and environmental clones in the RAxML (Randomized Accelerated Maximum Likelihood) program (Stamatakis et al., 2008) by using the GTRCAT substitution model. The bootstrap values were calculated from 1,000 replicates.

## Results

### Lake Chemistry

The water chemistry of the lake, previously described in Leboulanger et al. (2017), is characterized by high levels of Na^+^, K^+^, Mg^2+^, and Cl^-^, and low levels of Ca^2+^ (**Table 1**). It is noticeable that the concentration of Ca^2+^ in the lake water increased during the rainy season (Leboulanger et al., 2017) whereas the concentrations of Mg^2+^ stayed relatively constant.

**Table 1:** Dissolved anions, cations, silicon, and alkalinity in μM for October 2014 in Dziani Dzaha water.

T°C	pH	Alkalinity	Na^+^	K^+^	Cl^-^	Mg^2^	SO_4_^2-^	Si	Ca^2+^^∗^	H_2_S
31	9.18	258865	1016177	38608	892153	4724	2615	228	54	31


*^∗^Data missing for October 2014; this value is estimated with the average Ca concentration at the end of dry season in 2010, 2011, and 2015.*

Although values reported in the literature for the solubility product *K_sp_* of hydromagnesite at 25°C appear to disagree by as much as 10 log units (Gautier et al., 2014), a van’t Hoff interpolation of experimental *K_sp_* values recently reported by these authors at 25 and 50°C yields log *K_sp_* = -37.5 ± 0.5 at 30°C for the reaction

$${Mg}_{5}{\left({CO}_{3}\right)}_{4}{\left(OH\right)}_{2}\cdot 4H_{2}O=5{Mg}^{2+}+4{CO}_{3}^{2-}+2{OH}^{-}+4H_{2}O$$

Values calculated in the present study for the logarithm of the ion activity product range between log *Q* = -35.74 and log *Q* = -37.50, the corresponding saturation index ranging between Ω = 0 and Ω = 1.73 and suggesting that the lake waters are at equilibrium or slightly supersaturated with respect to hydromagnesite. The solubility product of aragonite at 30°C is log *K_sp_* = -8.37 (Plummer and Busenberg, 1982). The calculated ion activity products range between log *Q* = -7.03 and log *Q* = -8.01, corresponding to saturation index values ranging between Ω = 0.36 and Ω = 1.34. Hence, the lake waters also appear to be close to equilibrium with or slightly supersaturated with respect to aragonite. Although the uncertainties on the solubility product of hydromagnesite do not allow to draw a definitive conclusion, it is possible that the lower concentrations of Ca^2+^ observed toward the end of the dry season would displace the equilibrium

$${4CaCO}_{3}+5{Mg}^{2+}+6H_{2}O={Mg}_{5}{\left({CO}_{3}\right)}_{4}({OH}_{2})\cdot 4H_{2}O+4{Ca}^{2+}+2H^{+}$$

toward the right, favoring the replacement of aragonite by hydromagnesite (Supplementary Figure S1A).

The lake waters are highly supersaturated with respect to talc and sepiolite (Supplementary Figure S1B), but these phases may not be representative of the material formed in the stromatolites.

### Stromatolite Morphologies and Textures

Stromatolites were detected in abundance all around the lake near the shores except on the East shore (**Figure 1**). Numerous distinct structures were detected and classified in two main categories: stromatolites with a hard cauliflower-like surface that we named cauliflower crust (**Figure 1**, Supplementary Figure S2, and Supplementary Table S1) and stromatolites with friable surfaces (**Figure 1**, Supplementary Figure S3, and Supplementary Table S1).

### The Cauliflower Texture Is Specifically Associated With the Presence of Alphaproteobacteria and Pleurocapsales

**FIGURE 2:**
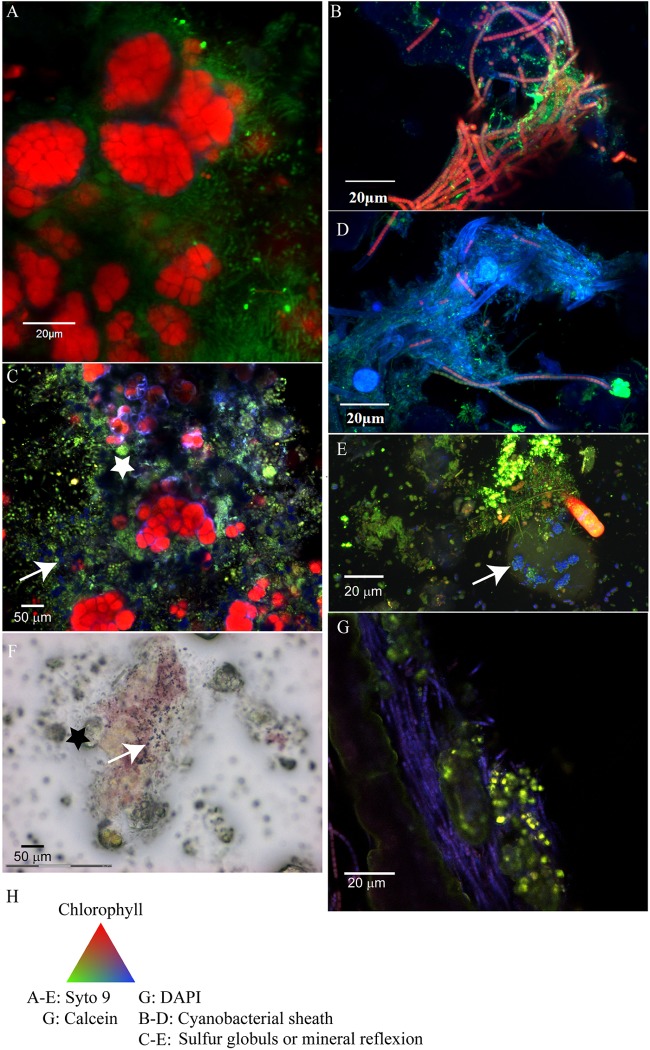
Confocal laser scanning microscopy (CLSM) and optical images of the microbial mats associated with the cauliflower-like crust **(A,C,F)** or columnar stromatolites with friable surfaces **(B,D,E,G)**. **(A)** Pleurocapsales (red) coated by filamentous microorganisms (green) located at the surface of the microbial mat associated with M17 fixed with formaldehyde and ethanol and stained with Syto9^®^ (green) observed with CLSM. **(B)** Filamentous Cyanobacteria (red) with persistent sheath (blue) detected by CLSM in the microbial mat associated with M12 fixed with formaldehyde and ethanol and stained with Syto9^®^ (the Cyanobacteria detected in M15 had the same morphology as in M12). **(C)** Microbial mat associated with M17 fixed with RNAlater^®^, stained with Syto9^®^ and observed with CLSM where micrometric coccoid microorganisms are visible in the empty sheath of Pleurocapsales (white star) and in association with refractive dots (blue, white arrow) which are probably elemental sulfur globules produced by the coccoid microorganisms. **(D)** Filamentous Cyanobacteria detected in the smooth surface of M10 which arbor shorter trichomes than Cyanobacteria detected on M12 and M15. **(F)** Microbial mat associated with M17 fixed with RNAlater and observed with optical microscopy in which elemental sulfur grains (black arrow) associated with the purple bacteria located around the Pleurocapsales cells (black star) are visible. **(E)** Red microbial mat associated with M15 fixed with RNA later and stained with Syto9^®^ in which refractive grains (blue, white arrow) in rod shaped bacteria probably corresponding to elemental sulfur globules are visible. **(G)** Transversal cutting of M10 stained with DAPI (blue) and calcein (green), included in LR white resin and observed with CLSM where aragonite grains stained by calcein (white arrow) trapped by filamentous Cyanobacteria stained with DAPI are visible. **(H)** Colors associated with the CLSM images.

The Cyanobacteria associated with the cauliflower crust exhibited characteristic shape of Cyanobacteria belonging to the Pleurocapsales order in the three samples observed (M16, M17, and biopile), i.e., colonies of coalescent globular cells with thick sheath mother cells and smaller daughter cells called baeocytes (**Figure 2**). The Pleurocapsales colonies were closely associated with 20 μm long filamentous microorganisms growing on their sheaths (**Figure 2**) in which electron transparent inclusions were detected, usually interpreted as poly-β-hydroxybutyrate deposits (D’Amelio et al., 1987; Tian et al., 2005) (Supplementary Figure S4). In areas where empty Pleurocapsales sheaths were present, due to Pleurocapsales cells degradation or the release of baeocytes, numerous morphologically different cells were also detected by Syto9^®^ staining (**Figure 2**). Most of them were small pink to purple cocci or rods able to form short chains growing in close association with the Pleurocapsales colonies and some invading the empty Pleurocapsales sheaths. These small pink cocci were sometimes associated with highly reflective particles, which are most probably composed of elemental sulfur globules (**Figures 2C,F**) that could be formed through the oxidation of sulfide by purple bacteria (Dahl and Prange, 2006). These highly reflective particles were detected only in samples fixed with RNAlater^®^, not in samples fixed with ethanol. Elemental sulfur globules indeed dissolve during ethanol dehydration and are often destroyed during usual EDS analyses (Dahl and Prange, 2006), we were thus unable to analyze them using EDS.

**FIGURE 3:**
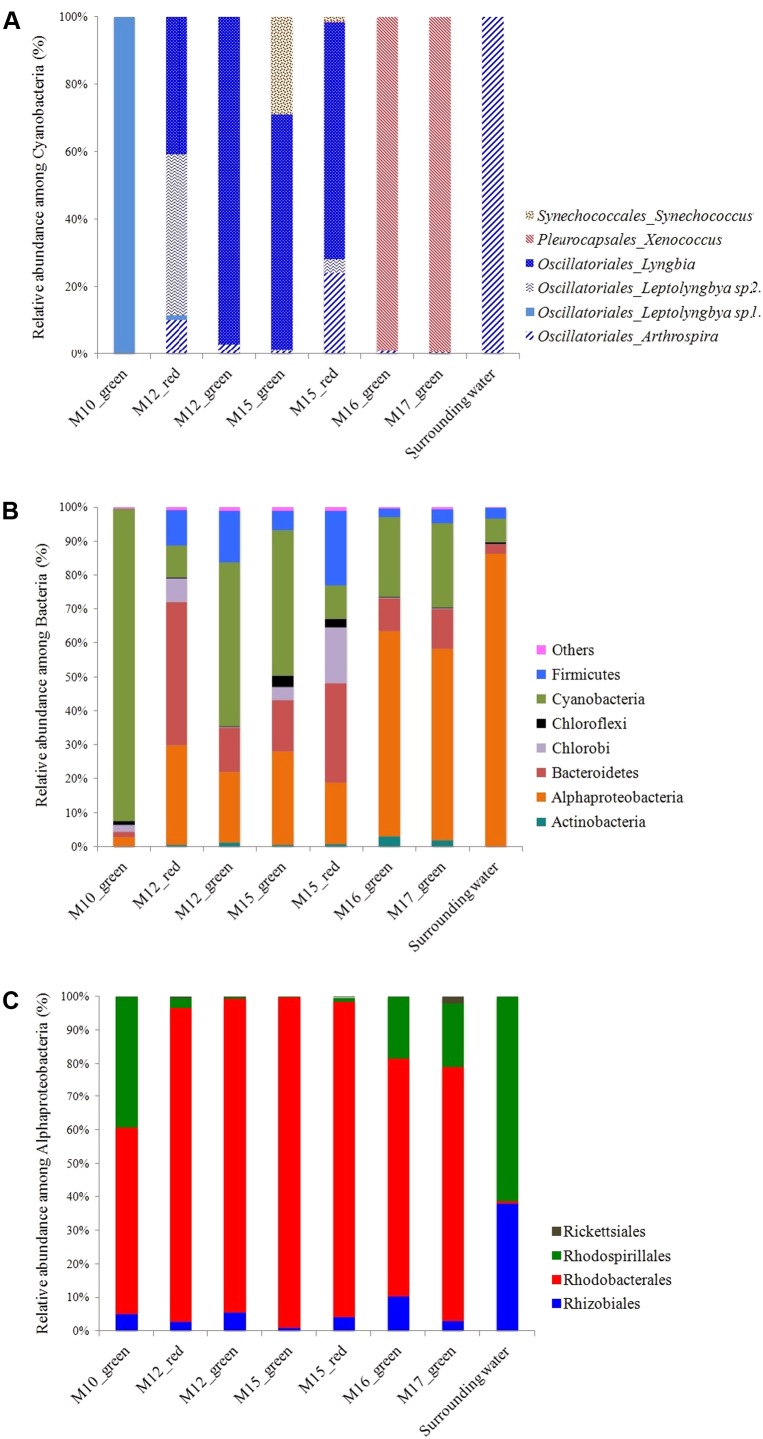
**(A)** Relative abundance of the different cyanobacterial classes inferred from 16S rRNA gene sequences in Dziani Dzaha stromatolites. **(B)** Proportion of sequences affiliated with major bacterial phyla (defined as >0.2% of total sequence number). **(C)** Relative abundance of the different alphaproteobacterial orders inferred from 16S rRNA genes sequences in Dziani Dzaha stromatolites.

The microbial community composition analyses based on 16S rRNA genes Illumina sequencing confirmed the observations made by CLSM. The rarefaction curves for the number of bacterial and archaeal OTUs detected in each sample indicated that a complete coverage of microbial composition was reached or nearly so (Supplementary Figure S5). Cyanobacteria affiliated to the Pleurocapsales order and to the *Xenococcus* genus were systematically and exclusively detected in cauliflower crusts of both stromatolites analyzed. They account for 99% and almost 100% of all the Cyanobacteria recovered in the DNA extract from the cauliflower crusts of the M16 and M17 stromatolites, respectively. Filamentous Cyanobacteria belonging to the *Leptolyngbya* genus were largely dominant in other stromatolites (M10, M12, and M15, **Figure 3**). However, filamentous Cyanobacteria belonging to the *Arthrospira* genus, dominant in the lake water, were not abundant in stromatolites. The maximal proportion of *Arthrospira* was retrieved in M15, in which they accounted for 24% of Cyanobacterial sequences.

The non-metric multidimensional scaling (NMDS) ordination of bacterial community including Cyanobacteria showed that M16 and M17 had similar bacterial community composition, distinct from those of the other stromatolites and of the lake water (Supplementary Figure S6). Alphaproteobacteria were largely dominant in M16 and M17 (around 60% of the bacterial sequences) and more abundant than Cyanobacteria (only 11 and 12% in M16 and M17, respectively). Alphaproteobacteria were also three times more abundant in M16 and M17 than in the other stromatolites. The most abundant Alphaproteobacteria detected in M16 and M17 belonged to the Rhodobacterales order and to the Rhodobacteraceae family (71 and 76% of the Alphaproteobacteria in M16 and M17, respectively) and mostly to several unknown genus (87 and 90% of Rhodobacterales, **Figure 3**). The second most abundant alphaproteobacterial family detected in M16 and M17 is Rhodospirillales *incertae sedis*, with bacteria belonging to the *Candidatus Alysiosphaera* genus (18 and 25% of all Alphaproteobacteria in M16 and M17, respectively). This genus comprises filamentous-flock-forming bacteria described from wastewater treatment plants, which are known to accumulate polyhydroxyalkanoate granules (Kragelund et al., 2006).

Alphaproteobacteria were also abundantly detected in stromatolites with friable surfaces (up to 27% of all bacterial 16S sequences detected in M15 green). They belonged mainly to the same unknown genus of the Rhodobacteraceae family as in M16 and M17, but they were also more diverse and encompassed species affiliated to *Roseibaca* (21–22% of the Rhodobacteraceae) which members produce bacteriochlorophyll a (Labrenz et al., 2009). Firmicutes belonging to the Clostridiales order were also detected in large amounts (up to 29% of all bacterial 16S sequences detected in M15 red), as well as Bacteroidetes (up to 22% of all bacterial 16S sequences detected in M12 red). Notably, filamentous Cyanobacteria stayed dominant in all the green parts of these stromatolites (92%, 45 and 30% in M10, M12 green and M15 green, respectively).

As observed for bacteria, archaeal communities detected in stromatolites were different depending on the presence or absence of the cauliflower crust. Archaea were detected in M17 but not in M16 samples. Haloalkaliphilic Archaea belonging to the Halobacteriales order and the *Natronococcus* genus constituted 98% of the archaeal sequences in M17. We recovered nine different classes of Archaea in M10, M12, and M15 (Supplementary Figure S7). This phylogenetic variety of Archaea was not detected previously in the microbialites of Alchichica Lake which represent a similar environment (Couradeau et al., 2011; Saghai et al., 2015).

Regarding eukaryotes, the presence of diatoms and green algae belonging to the *Picocystis* genus (data not shown) sometimes abundantly entombed was reported in all types of stromatolite using CLSM and SEM. Furthermore, patches of fly pupae were present (probably belonging to the Ephydridae family, common in brine waters) and their entombment contributes significantly to the stromatolites construction (Supplementary Figures S2, S3). For this study, only the surfaces of stromatolites devoid of fly pupae were analyzed.

### Mineral Composition of Stromatolites

The bulk analysis of six different stromatolites by XRPD revealed that they were dominated by aragonite together with small amounts of hydromagnesite, halite, calcite, Mg-calcite and dolomite (Supplementary Figure S8). Hydromagnesite was only present in the cauliflower crusts where the consortia of Pleurocapsales and Alphaproteobacteria were found. In one occurrence 27% of albite was also detected, which we interpret as a detrital input incrusted in the stromatolite. Halite most probably results from the drying of the pore water since stromatolites were not rinsed in distilled water prior to XRPD analyses.

### Minerals/Microorganisms Interactions

#### In the Cauliflower Crust

Using CLSM, Raman and SEM, three distinct mineral phases were observed in the cauliflower crust: aragonite, hydromagnesite and a magnesium silicate phase probably poorly crystallized, since it was not detected by XRPD.

**FIGURE 4:**
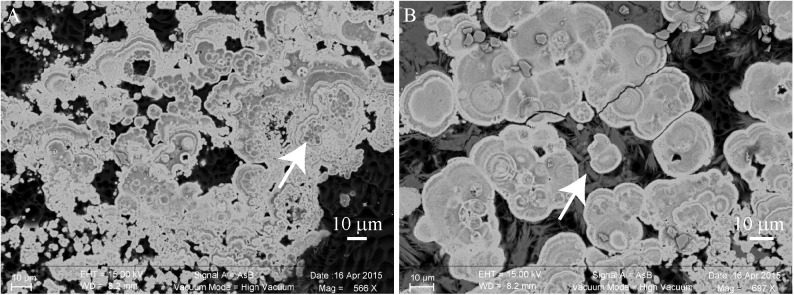
Scanning electron microscopy (SEM) images of a transversal cutting of the cauliflower-like crust from a pink colored area of M17, embedded in LR white resin, showing the mineral distribution in the microbial mat. **(A)** Relics of micrometric cells (white arrow) that seemingly served as nucleation points for aragonite (bright gray) precipitation. **(B)** Hydromagnesite needles (dark gray, white arrow) growing on aragonite grains.

**FIGURE 5:**
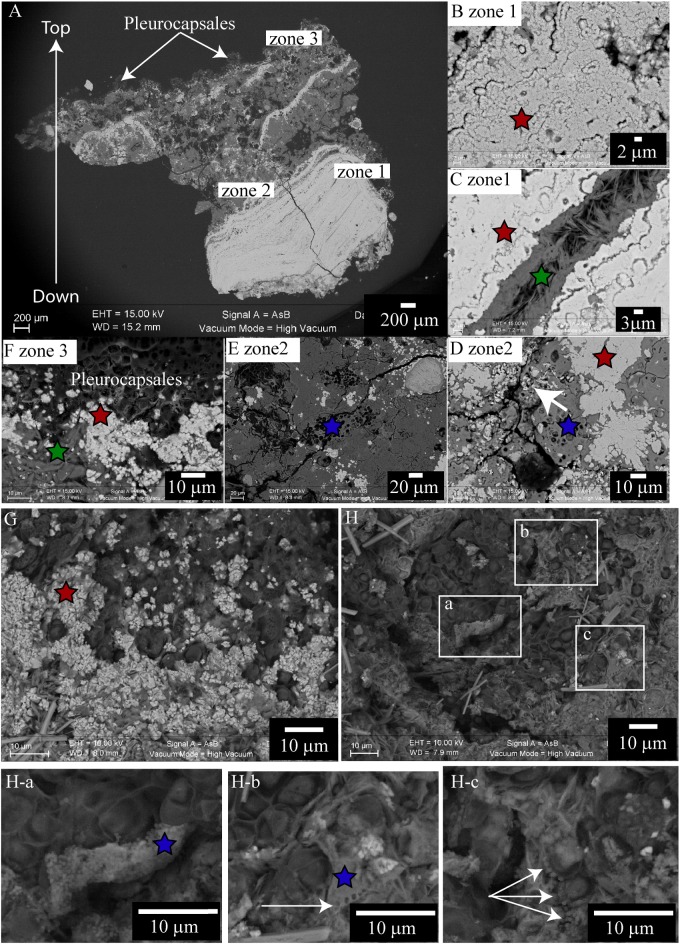
Scanning electron microscopy images of a transversal cutting of the cauliflower-like crust from a green/pink colored area of M17, embedded in LR white resin **(A–F)**, or top of the microbial mat of M17 desiccated to the critical point **(G,H)**, showing the mineral distribution in the microbial mat. In all the images red stars correspond to aragonite; green stars correspond to hydromagnesite; blue stars to magnesium silicate. **(A)** Global view of the globular crust (at 15 KV, AsB detector), bright gray indicates the Ca rich phases and dark gray the Mg rich phases. Colonies of Pleurocapsales are visible on the top of the transversal cutting. **(B–F)** Enlargement of the transversal cutting shown in **(A)**. **(B)** Aragonite zone at the bottom of the transversal cutting with relics of micrometric cells (at 15 KV, AsB detector). **(C)** Hydromagnesite needles grown on aragonite (at 15 KV, AsB detector). **(D)** Aragonite grown on relics of Pleurocapsales cells embedded in a magnesium silicate phase (at 15 KV, AsB detector). **(E)** Relics of Pleurocapsales colonies embedded in a magnesium silicate phase (at 15 KV, AsB detector). **(F)** Aragonite precipitated at the bottom of Pleurocapsales colonies and hydromagnesite needles on aragonite (at 15 KV, AsB detector). **(G)** Aragonite precipitated around Pleurocapsales cells at the surface of the microbial mat (at 10 KV, AsB detector). **(H)** Pleurocapsales embedded in a magnesium silicate phase (at 10 KV, AsB detector). **(H-a – H-c)** Enlargements of the SEM image shown in **(H)** highlighting Mg-Si beads around the Pleurocapsales cells. White arrow in **(H-b)** indicates holes in the silicate magnesium phase. White arrows in **(H-c)** indicate silicate beads around Pleurocapsales cells.

**FIGURE 6:**
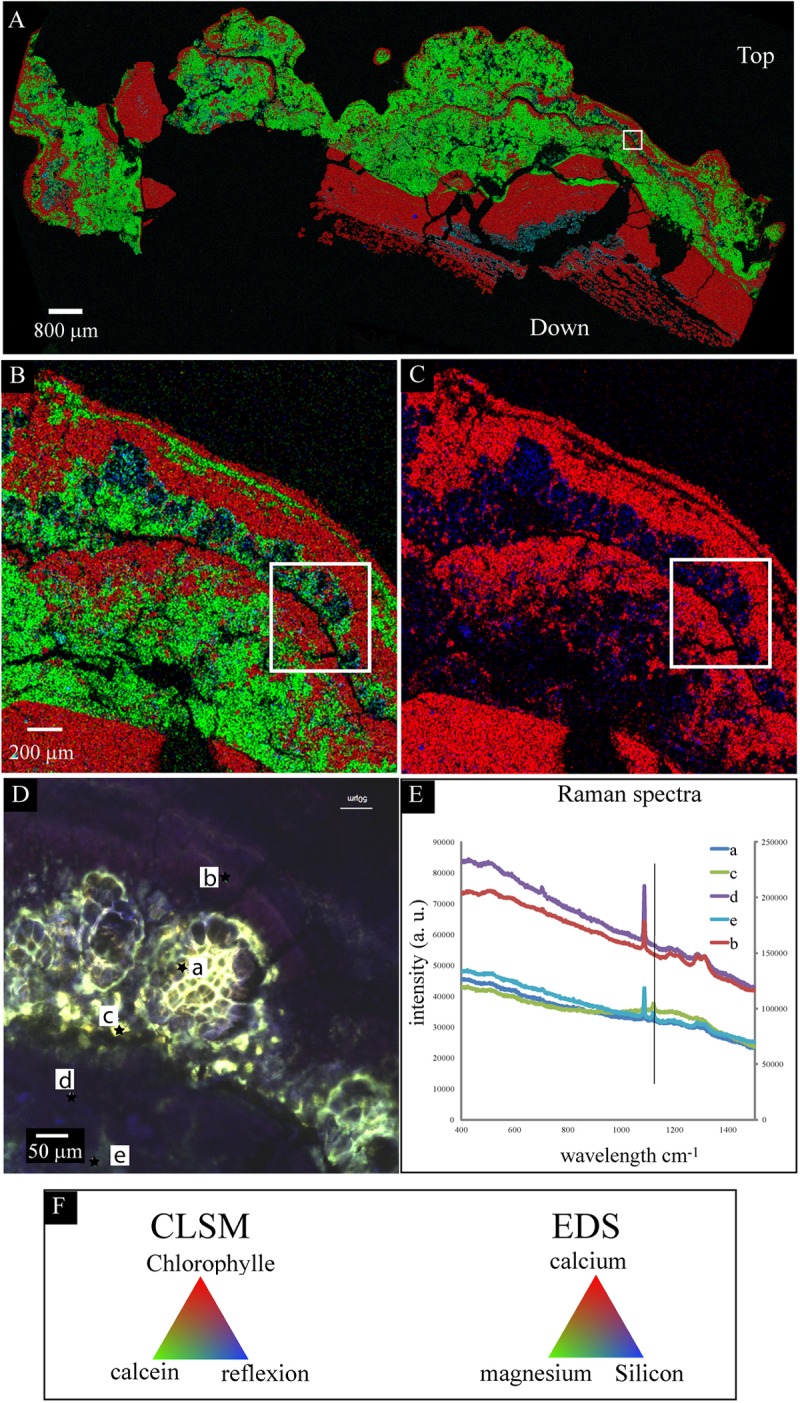
Scanning electron microscopy-EDS/CLSM/Raman on the same transversal cutting of the globular crust of M16 embedded in LR white resin, showing the accumulation of silicon in the sheaths of Pleurocapsales. **(A,B)** SEM-EDS mappings of calcium, magnesium, and silicon. **(C)** SEM-EDS mapping of calcium and silicon in the same area as in **(A,B)**. **(B,C)** Are enlargement of the mapping shown in **(A)**. The white rectangle shows exactly the same zone in the four images **(A–D)**. In **(A–C)**, the EDS mapping shows that calcium and silicon were negatively correlated whereas magnesium and silicon were sometimes positively correlated. **(D)** CLSM image of the same area showing that the silicon accumulated in the empty sheaths of Pleurocapsales. **(E)** Raman spectra of points a, b, c, d, and e highlighted in **(D)**. The vertical line fits the specific peak for hydromagnesite detected at points a, e, and c. The other peaks are specific for aragonite that was detected at all points. **(F)** Colors associated with the CLSM **(D)** and EDS mappings **(A–C)**.

**FIGURE 7:**
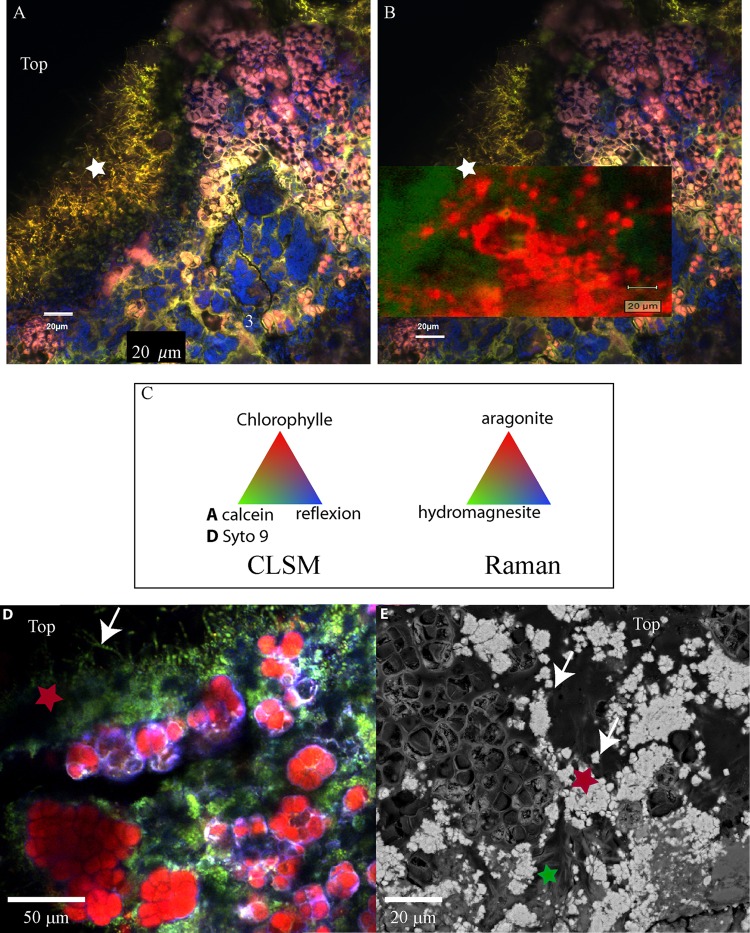
**(A)** Confocal laser scanning microscopy image of a transversal cutting of the globular crust of M17 embedded in LR white resin, showing the Pleurocapsales colonies (pink/red) topped with grains stained with calcein (green) and filamentous Alphaproteobacteria also strongly stained with calcein (green, white star). **(B)** Raman mapping superimposed on the CLSM image showing the distribution of the aragonite grains around the Pleurocapsales colonies. **(C)** Colors associated with the CLSM and Raman mappings. **(D)** CLSM image of the microbial mat fixed in RNAlater and stained with Syto9 (green) showing the distribution of the Pleurocapsales colonies topped with coccoid microorganisms associated with aragonite grains and filamentous cells on the top. **(E)** SEM image (at 15 KV, AsB detector) on the same transversal cutting shown in **(A,B)**, showing aragonite grains associated with the coccoid and filamentous microorganisms associated with the Pleurocapsales. Red stars correspond to aragonite and the green star to hydromagnesite in all images.

This millimetric cauliflower crust was always associated with an endolithic intermixed green and purple microbial mat (Supplementary Figure S2F). This microbial mat developed also above the crust (Supplementary Figures S2E,F). Below the water surface, the crust was pink pigmented. In some zones, the green biofilm developed above the pink crust, while in some others, the pink crust developed above the green mat and fly pupae (Supplementary Figure S2E). This suggests that the green mat and the pink crust grew up alternatively (Supplementary Figure S2E). In this pink crust, aragonite was the main mineral phase detected, with spikes of hydromagnesite growing on it (**Figure 4**). Relics of micrometric coccoid cells which have been used as nucleation sites for aragonite precipitation were detected in the center of aragonite grains (**Figure 4A**). No relics of Pleurocapsales were found in the pink crust, this suggests that the pink aragonite crust grew only in association with the pink pigmented Alphaproteobacteria. Indeed, Alphaproteobacteria are the most abundant microorganisms detected in the 16S RNA gene sequences retrieved from the cauliflower crusts. Below the green microbial mat (Supplementary Figures S2E,F), where Pleurocapsales were detected, aragonite laminations of a few microns were identified in the first hundreds of microns of the upper crust, alternating with hydromagnesite mixed with a silicon rich phase (**Figures 5**–**7** and Supplementary Figures S9, S12). In this phase, embedded relics of Pleurocapsales colonies were observed (**Figures 5**, **6** and Supplementary Figure S12). The EDS analyses of this phase indicated a Mg-silicate phase (Supplementary Figure S10) with a Mg/Si average ratio of 0.90. The repartition of silicon and magnesium in the chemical maps of stromatolites showed that this phase, abundant in the cauliflower crust (**Figure 6** and Supplementary Figures S9, S12), was related to the presence of the Pleurocapsales colonies. Silicon accumulation was evidenced in the sheaths of Pleurocapsales, correlated with magnesium in several occurrences (**Figure 6** and Supplementary Figure S12). This accumulation increased from the top to the bottom of the mat seemingly coinciding with the decay of the mat. Preserved Pleurocapsales sheaths with the silicate phase were identified in M16 below 200 μm layers of aragonite and hydromagnesite, but the sheaths were empty without any photosynthetic pigment detectable (**Figure 6**). Using SEM, formation of submicrometric magnesium-and-silicon rich beads around the sheaths of Pleurocapsales were furthermore identified (**Figure 5** and Supplementary Figure S11).

The SEM-EDS maps showed that calcium was negatively correlated with silicon and magnesium (**Figure 6** and Supplementary Figures S9, S12). At the top of the mat, aragonite particles were detected by SEM and Raman spectroscopy around Pleurocapsales colonies, where coccoid microorganisms were also detected by CLSM (**Figure 7**). Scarce aragonite particles were also detected in some Pleurocapsales cells that were probably decaying. The precipitation of aragonite thus seems to be favored by coccoid microorganisms which may also be at the origin of the micrometric organic globules detected in the pink aragonite crust (**Figure 4**).

Raman and CLSM analyses showed in addition that some aragonite particles were clustered around the filamentous Alphaproteobacteria at the top of the mat (**Figure 7**). It is noticeable that these filamentous bacteria were strongly stained with calcein, revealing the presence of calcium or magnesium (**Figure 7**). Raman cartography showed that they were also associated with hydromagnesite (**Figure 7**).

More generally, SEM and Raman spectroscopy measurements were consistent with the growth of hydromagnesite needles on aragonite particles at the top of the green mat or in the pink crust (**Figures 4**, **7**). Underneath the cauliflower crust, in the deepest part of stromatolites, aragonite was the only mineral detected in friable laminations (Supplementary Figures S2F, S9); both hydromagnesite and the Mg-Si phase were absent.

#### In the Columnar Stromatolites With Smooth and Granular Friable Surfaces

**FIGURE 8:**
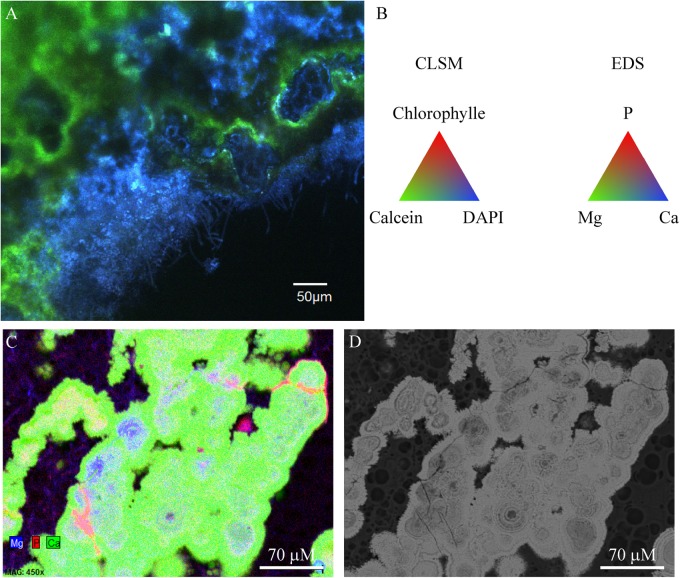
Confocal laser scanning microscopy/Raman/SEM-EDS on the same transversal cutting in red colored zone of M12 microbial mat embedded in LR white showing aragonite precipitating around the microbial mat. **(A)** CLSM image showing coccoid, rod shaped and filamentous microorganisms (blue) being incrusted in aragonite (green). No Cyanobacteria were detected in this zone. **(B)** Colors associated with the CLSM and EDS mappings. **(C)** EDS mapping of the same transversal cutting showing the centers of aragonite grains where magnesium and phosphorus is detected, which could correspond to relics of the encrusted microbial mat. **(D)** SEM image of the same image as in **(C)** (at 15 KV, AsB detector). The LR white resin appears in black and aragonite in bright gray.

Aragonite grains were mainly trapped by filamentous Cyanobacteria (**Figure 2**), or massive aragonite precipitation and entombment of the whole biofilms were observed in areas devoid of Cyanobacteria (**Figure 8**). This resulted in purple and green laminations of aragonite of variable densities and coloration (Supplementary Figure S3F). The transversal cutting of the aragonite grains at the surface of stromatolites showed that their center was sometimes enriched in magnesium in M10, M12, and M15 (**Figure 8**). This magnesium enrichment in the center of the aragonite grains was also detected in the deepest parts of M17 and M16 under the cauliflower crusts (not shown), suggesting that they could have been formed previously by the same type of community as observed at the surface of M10, M12, or M15. The only mineral phases containing magnesium detected in the friable stromatolites surfaces were magnesian calcite and dolomite, both in small amounts (Supplementary Figure S8). A morphological correlation can be done between the whole biofilm embedded in aragonite and the center of the aragonite grains rich in magnesium (**Figure 8**), suggesting that the magnesium accumulation could be linked to the presence of the entombed microorganisms. No magnesium silicate phase was detected by SEM.

### Microdissection and Whole Genome Amplification

In order to gain more information on the phylogeny and metabolic capabilities of the microorganisms associated with the cauliflower crusts, laser microdissection was used to isolate consortia of Pleurocapsales with closely associated coccoid microorganisms (Supplementary Figure S13). The coccoid microorganisms were pink to purple pigmented (**Figure 2**). After whole genome amplification and 16S RNA gene analysis, one species of Pleurocapsales (in five different amplifications) and one species of Rhodobacteraceae (in two different amplifications) were identified.

The Pleurocapsales species shares 98% 16S rRNA gene identities with one Pleurocapsales species associated with the microbialites of Alchichica Lake in Mexico (accession number JN825323). However, this Pleurocapsales species was not the main calcifying Pleurocapsales (accession number JN825326) detected in the Alchichica microbialites (Supplementary Figure S13). The Dziani Dzaha Pleurocapsales is also closely related to Pleurocapsales species belonging to the microbial community involved in marble dissolution (accession numbers JQ404416 and JQ404419) (Supplementary Figure S13). The Pleurocapsales detected in Dziani Dzaha stromatolites could thus have the capacity of colonizing and dissolving calcium carbonate. However, we did not detect clear evidence of Pleurocapsales boring activity in the samples collected.

Interestingly, we found that the microdissected Rhodobacteraceae is also closely related to bacteria of the same microbialites in Alchichica lake (JN825344) (Supplementary Figure S14), suggesting that this type of Rhodobacteraceae may have an important role in the formation of microbialites not only in lake Dziani Dzaha. The Rhodobacteraceae of Dziani Lake and Alchichica Lake microbialites share 98% identities at the level of their 16S rRNA gene (Couradeau et al., 2011). They are also closely related to Alphaproteobacteria detected in the extreme saline-alkaline soil of the former lake Texcoco (Mexico) (accession numbers JN178563 and JN177885). They are distantly related (94% identities) to known cultivated species such as *Amaricoccus* species isolated from activated sludge system (Falvo et al., 2001) and phototrophic purple non-sulfur bacteria such as *Rhodobacter blasticus* (Eckersley and Dow, 1980).

## Discussion

The presence of the three mineral phases (aragonite, hydromagnesite, Mg-silicate) closely associated with microorganisms at the top of the mat in the globular crust is in accordance with geochemical modeling taking into account the chemistry of lake water. This was modeled precisely in the case of aragonite and hydromagnesite (Supplementary Figure S1A). The unknown thermodynamic properties of the precipitated Mg-silicate phase prevent such a modeling; however, the formation of this phase is still consistent with thermodynamic modeling which predicts the formation of talc and sepiolite (Supplementary Figure S1B), the precipitation of which appears to be kinetically hindered in the conditions of precipitation. Instead, a metastable Mg-silicate yet to be characterized is precipitated in local microenvironments provided by some microorganisms of the stromatolites.

### Hydromagnesite Precipitation

Hydromagnesite was detected on the aragonite grains suggesting that it precipitated preferentially with respect to aragonite in calcium-depleted microenvironments as shown by the stoichiometry of reaction (1). However, hydromagnesite was only found at the top of the cauliflower crust where the phototrophic pigmented microorganisms were detected and not in the stromatolites having a friable surface, suggesting that its precipitation could be biologically influenced. Indeed, hydromagnesite was more particularly associated with the filamentous yellow pigmented Alphaproteobacteria at the top of the mat. These bacteria were systematically located at the surface of the microbial mat (**Figure 7** and Supplementary Figure S4), suggesting that their growth may be light-dependent, as could be the accumulation of polyhydroxyalkanoate granules, the location of which has been detected by SEM. The formation of polyhydroxyalkanoate through the phototrophic activity of purple non-sulfur bacteria (PNSB) has been well documented (Liebergesell et al., 1991; Higuchi-Takeuchi et al., 2016). Furthermore, as revealed by calcein staining, these filamentous bacteria accumulated Ca^2+^ and/or Mg^2+^ in their sheaths. This feature could be related to the capacity of this kind of filamentous cells to form flocks where bacteria are strongly bound to each other. Indeed, it has been shown that calcium and to a lesser extent magnesium may be necessary in the process of bacterial cells adhesion (Kragelund et al., 2006). These filamentous cells may thus potentially change the Mg/Ca ratio at the surface of the mat and could have an influence on the balance between aragonite or hydromagnesite precipitation. In addition, the adhesion properties of these bacteria probably played a key role in the trapping and binding of hydromagnesite spikes as well as aragonite particles. It has already been shown that Cyanobacteria isolated from the alkaline lake Salda (SE Turkey) mediate the precipitation of hydromagnesite (Shirokova et al., 2013). However, it is not clear at this stage if the metabolic activity of these potentially photosynthetic filamentous Alphaproteobacteria induced the precipitation hydromagnesite in Dziani stromatolites. Further experiments should be carried out in the laboratory to answer this question, if it is possible to isolate and cultivate these bacteria.

### Mg-Silicate Precipitation

The formation of the Mg-silicate seemed to be initiated by the accumulation of Si and Mg in the sheaths of Pleurocapsales where the formation of microbeads of Mg-Si was detected. Similar microbeads were observed in association with the decay of the EPS from Cyanobacteria belonging to the *Synechococcus* genus in the ocean (Tang et al., 2014). The authors suggested that the progressive accumulation of magnesium and silicon in decaying EPS of *Synechococcus* species leads to the formation of these beads that could account for a significant part of the silicate found in the bottom of the ocean (Tang et al., 2014). Similarly, the accumulation of silicon and magnesium in the Pleurocapsales sheaths during their decay may be at the origin of the Mg-Si phase observed in the cauliflower crust of stromatolites. The presence of Mg-Si phases was already reported in several modern lacustrine microbialites: in Mono Lake and Great Salt Lake (United States), in the Crater Lake Satonda (Indonesia), in basaltic caves at Kauai (Hawaii), in the alkaline and hyposaline Lake Clifton in Western Australia, and in several Mexican crater lakes in Mexico (Léveillé et al., 2000a,b, 2002, 2007; Arp et al., 2003; Souza-Egipsy et al., 2005; Burne et al., 2014; Zeyen et al., 2015; Pace et al., 2016). Interestingly, Mg-silicates associated with fossil microbialites have also been reported in the extensive hydrocarbon-bearing Pre-Salt layer offshore of Brazil (Corrêa, 2012) and Angola (Wasson et al., 2012), which were interpreted as the result of lacustrine deposition in highly alkaline lake waters (Wright, 2012). Furthermore, Mg-silicates seem to be important in the formation of microbialites: the modern thrombolites of Lake Clifton, Western Australia, for example, gain their initial structural rigidity from biofilm mineralization with Mg-silicate (Burne et al., 2014). Similarly, Mg-silicate is associated with the most rigid surface texture observed on Dziani stromatolites. However, in modern microbialites, Mg-silicates seem to be unstable and are rapidly replaced by aragonite following the degradation of the associated organic components (Burne et al., 2014; Pace et al., 2016). Although the Mg-silicate was only detected in association with Pleurocapsales sheaths in the cauliflower crust, its replacement by aragonite was not clearly evidenced. Nevertheless, it suggests that the Mg-silicate was dissolved in the deepest part of the stromatolites. An alternative explanation would be that the internal part of the stromatolites building was mediated by another type of microbial consortium, as the one observed in the friable surface stromatolites dominated by filamentous Cyanobacteria, and that the Mg-silicate never formed.

### Aragonite Precipitation

In the cauliflower crust, Rhodobacteraceae were associated with the precipitation of aragonite. Given the pink to purple pigmentation of the cells and the pink coloration of the aragonite crust, these Rhodobacteraceae coccoid cells were potentially phototrophic bacteria. Indeed, the Rhodobacteraceae family comprises, among others, purple non-sulfur bacteria (PNSB), which possess an extensive range of metabolic capabilities (Pujalte et al., 2014). PNSB may accumulate sulfur globules outside their cells during anoxygenic photosynthesis based on the oxidation of reduced sulfur compounds (Dahl and Prange, 2006). Probable sulfur globules were detected with CLSM, indicating that some of the Alphaproteobacteria were oxidizing reduced sulfur compounds. This is in accordance with the relatively high level of H_2_S (31–100 μM between the surface and 1 m depth) measured in October 2014. Some coccoid cells colonized empty Pleurocapsales sheaths suggesting as well that photoheterotrophic or heterotrophic activities could have induced the formation of aragonite. Anoxygenic photoautotrophic bacteria are supposed to favor precipitation of calcium or magnesium carbonates through alkalization during photosynthesis (Dupraz and Visscher, 2005; Visscher and Stolz, 2005). However, it has also been shown that anoxygenic photosynthesis in which H_2_S is oxidized to S° may, in a second step, acidify the medium when S° is ultimately oxidized to sulfate in presence of oxygen (Visscher and Stolz, 2005). Comparatively, photoheterophy may be a more efficient alkalinizing process leading to the precipitation of calcium carbonate in PNSB environment (Bundeleva et al., 2012), depending on the organic substrate used. It is thus not possible to know exactly at this stage which metabolism favored aragonite precipitation.

Other bacterial lineages could have influenced aragonite formation although we did not evidence that using microscopic or spectrometric methods. Firmicutes belonging to the Clostridiales order were present in all stromatolites and represented an important proportion of the microbial composition associated with M15 and M12 stromatolites. Members of the Clostridiales order encompassed SRB (Castro et al., 2000) and that type of metabolism has been shown to induce aragonite formation as a result of the degradation of Cyanobacteria and extracellular organic matrix (Pace et al., 2016). Furthermore, members of the Chlorobi and Chloroflexi bacterial lineages, that encompass anoxygenic photosynthesizers (Bryant and Frigaard, 2006), were abundantly detected in M15, M12, and M10 stromatolites and could also have participated in the formation of aragonite. Thus, we cannot exclude that SRB, Chlorobi and Chloroflexi could have favored the formation of aragonite and we should continue to investigate this possibility in particular in the stromatolite M15, where they were the most abundantly detected.

### Influence of Microorganisms on the Stromatolites Surface Textures

Different types of Cyanobacteria were associated with different stromatolite surface textures. Oscillatoriales, trapping and binding aragonite grains, were associated with friable surfaces, whereas in the hard cauliflower surfaces, we observed the permineralization of Pleurocapsales by Mg-silicate. The difference in surface textures may be linked to the locus of mineral precipitation. Mg-silicate precipitation takes place within the sheaths of Pleurocapsales which form dense colonies, while no evidence of mineral precipitation was detected in the sheaths of Oscillatoriales. The rigidity of the cauliflower crusts may be due to the presence of the Mg-silicate phase as previously noticed in the microbialites of Lake Clifton [Western Australia (Burne et al., 2014)]. The property of Pleurocapsales to favor mineral precipitation in their sheaths has already been highlighted in the microbialites from Lake Alchichica in Mexico (Couradeau et al., 2013) and in several other lacustrine and marine microbialites, such as microbialites from Lake Van, Turkey (Kempe et al., 1991; Lopez-Garcia et al., 2005), Satonda, Indonesia (Arp et al., 2003) and the Bahamas (Mobberley et al., 2012), except that aragonite was precipitated with Pleurocapsales in these cases (Couradeau et al., 2013). This may be due to different sheath properties or different environmental conditions, and in particular to variation of dissolved silica concentrations. Indeed, we detected a calcium accumulation in the sheath of Pleurocapsales in the microbialites of Alchichica Lake (Gerard et al., 2013) but not in the sheath of the Pleurocapsales of Dziani Dzaha stromatolites. However, the concentration of calcium is five time higher in Alchichica Lake water than in Dziani Dzaha Lake water [54 μM in Dziani Dzaha Lake and 275 μM in Alchichica Lake (Armienta et al., 2008)] and the concentration of ortho-silicic acid is less than 160 μM in Alchichica Lake where no magnesium–silicate phase was observed. Nonetheless, it emphasizes the important role of Pleurocapsales in the formation of lacustrine and marine microbialites (Zeyen et al., 2017). In addition, the high abundance of calcifying Alphaproteobacteria in the cauliflower crusts must also have played a role in the stiffening of the structure.

## Conclusion

Purple non-sulfur bacteria and Cyanobacteria influenced the shape and mineralogy of the Dziani Dzaha stromatolites. The formation of Mg-silicate was biologically mediated by the presence of Pleurocapsales. Purple non-sulfur bacteria were the dominant and the most calcifying microorganisms in the microbial mats associated to stromatolites with a cauliflower shape. They fossilized into aragonite and contributed to the formation of aragonite and hydromagnesite laminations. As stromatolites developed under an anoxic Earth surface before 2,500 Myr, this raises the possibility that the oldest stromatolites were formed by microbial communities dominated by anoxygenic phototrophic bacteria. To test this idea, it will be necessary to analyze the metabolic capacities of the purple non-sulfur bacteria involved in aragonite precipitation through the analysis of their genome after laser microdissection and whole genome amplification to determine if they can be primary producers in these microbial mats. Attempts to isolate them in pure culture should be done to measure their capacity to induce carbonate precipitation without the influence of Cyanobacteria. Furthermore, it would be interesting to follow the evolution of the microbial communities and mineral composition associated with stromatolites according to the lake water level, as some of the stromatolites analyzed may be placed at the oxic/anoxic transition zone when the lake is at its highest level. These conditions may be more representative of the Precambrian conditions with low level or absence of oxygen.

## Author Contributions

EG, SD, MH, HA, LR, VM, FG, LL, SB, TL, M-BJ, GS, and DJ made the analyses. EG wrote the manuscript. EG, SD, LR, FG, MH, HA, VM, GS, CL and MA discussed the results and revised the manuscript. EG, MA, CL, HA, DJ, and GS performed the Dziani Dzaha sampling expedition and provided the stromatolites. MA and CL obtained funding for the project. All authors read and approved this manuscript.

## Conflict of Interest Statement

The authors declare that the research was conducted in the absence of any commercial or financial relationships that could be construed as a potential conflict of interest.

## Footnotes

1 http://cran.r-project.org/web/packages/vegan/index.html
