# Supplementary material for: Key Role of Alphaproteobacteria and Cyanobacteria in the Formation of Stromatolites of Lake Dziani Dzaha (Mayotte, Western Indian Ocean)
Source: Front Microbiol. 2018 May 22;9:796. doi: 10.3389/fmicb.2018.00796 (PMC5972316; doi:10.3389/fmicb.2018.00796)
Supplement: Supplementary file 1 [file Data_Sheet_1.pdf]

Samples	Longitude E	Latitude S	Surface	Cyanobacteria type (CLSM)
M10	45°17'16.30"	12°46'19.25"	Smooth friable	Oscillatoriales
M12	45°17'16.30"	12°46'19.25"	Granular friable	Oscillatoriales
M14 (analog M15)	45°17'08.84"	12°46'15.53''	Granular friable	Oscillatoriales
M15	45°17'08.84"	12°46'15.53''	Granular friable	Oscillatoriales
M16	45°17'15.89"	12°46'8.38"	Cauliflower-like crust	Pleurocapsales
M17	45°17'15.89"	12°46'8.38"	Cauliflower-like crust	Pleurocapsales
biopile	45°17'21.47"	12°46'18.37"	Cauliflower-like crust	Pleurocapsales
M19 (fossil)	45°17'15.89"	12°46'8.38"	Cauliflower-like crust	ND
Point 18 m	45°17'23.22"	12°46'14.88"		

Table S1: GPS coordinates of the stromatolites sampled in Dziani Dzaha and of the point 18 m where water was collected for chemical analyzes. The texture and type of Cyanobacteria observed by CLSM are indicated for each stromatolite collected.

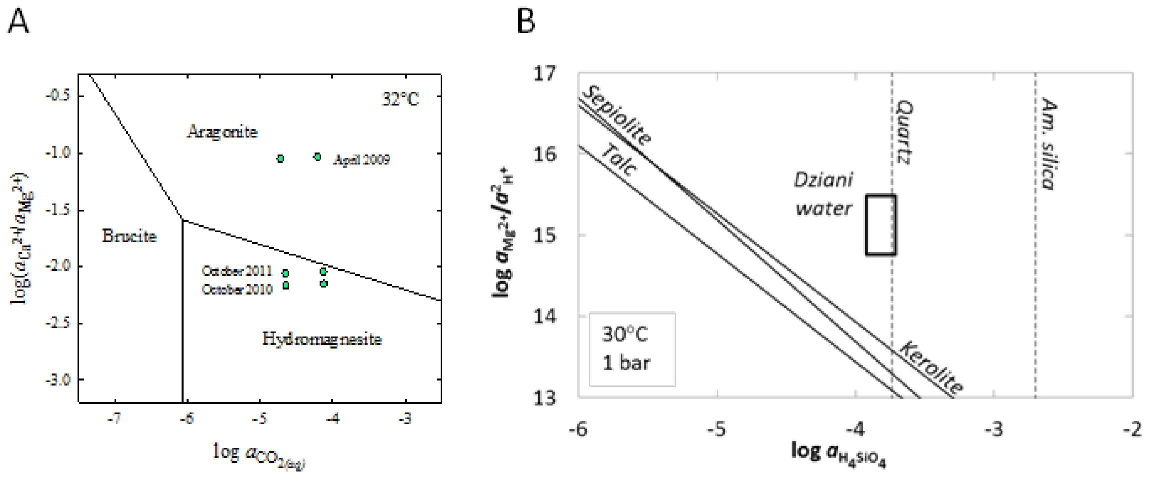

Figure S1: A) Phases diagram of the system CaO-MgO-CO_2 -H_2O. B) Solubility diagram for the Mg-silicate system (MgO-SiO2-H2O) at 30°C and 1 bar. The equilibrium solubilities for sepiolite, talc and kerolite are indicated with solid lines. Vertical dashed lines are the equilibrium solubilities of quartz and amorphous silica. The black square represents the range of water compositions measured by Leboulanger et al; 2017 and Milesi et al. (submitted to Sedimentology). Thermodynamic data are from the Thermoddem database (Blanc et al. 2012) for sepiolite, talc, quartz and amorphous silica , and from (Stoessel 1988) for kerolite. The stability domain of kerolite is calculated at 25°C.

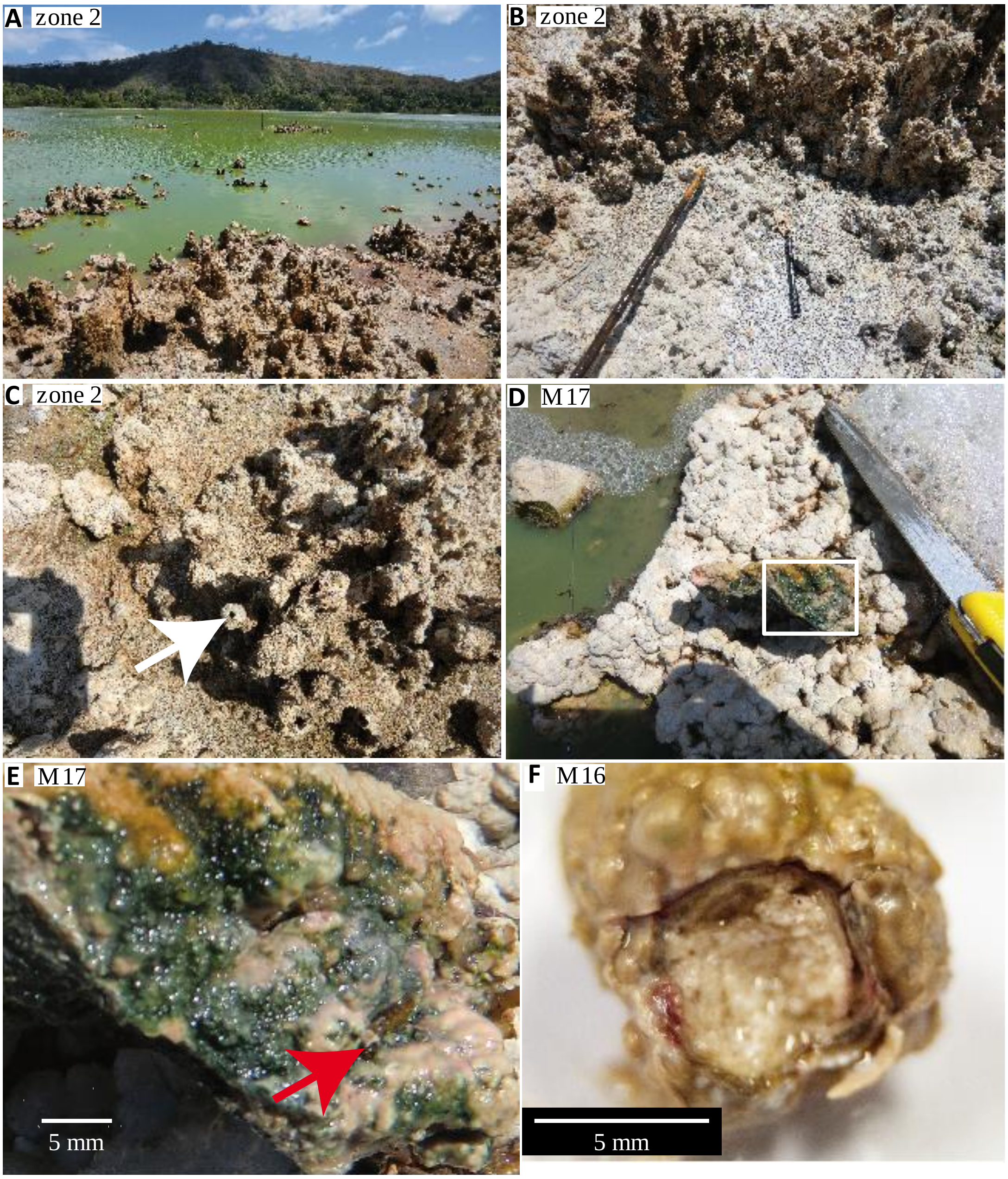

Figure S2: pictures of stromatolites with a cauliflower-like crust at the surface. At the time of collection, clumps of emerged dome-shaped and small columnar stromatolites, immerged during the wet season, were visible (Panels A, B, C, D). A white cauliflower-like hard structure developed horizontally (panels B and D) and formed columns of few decimeters high. Some of these columnar stromatolites were empty (white arrow in panel C), attesting that processes of dissolution have probably occurred internally. Just below the water surface a green microbial mat associated with a pink crust developed on these cauliflower surfaces, the pink crust growing sometimes above fly pupae (red arrow in panel E). Numerous bubbles, probably oxygen due to active photosynthesis, were visible in association with the green areas. The transversal cutting of the millimetric cauliflower texture revealed a thin layer of green and purple microbial mat in all cauliflower-like crust (panel F). Below this thin layer of green-purple microbial mat, a less organized, seemingly partially dissolved mineral structure was always observed (panel F).

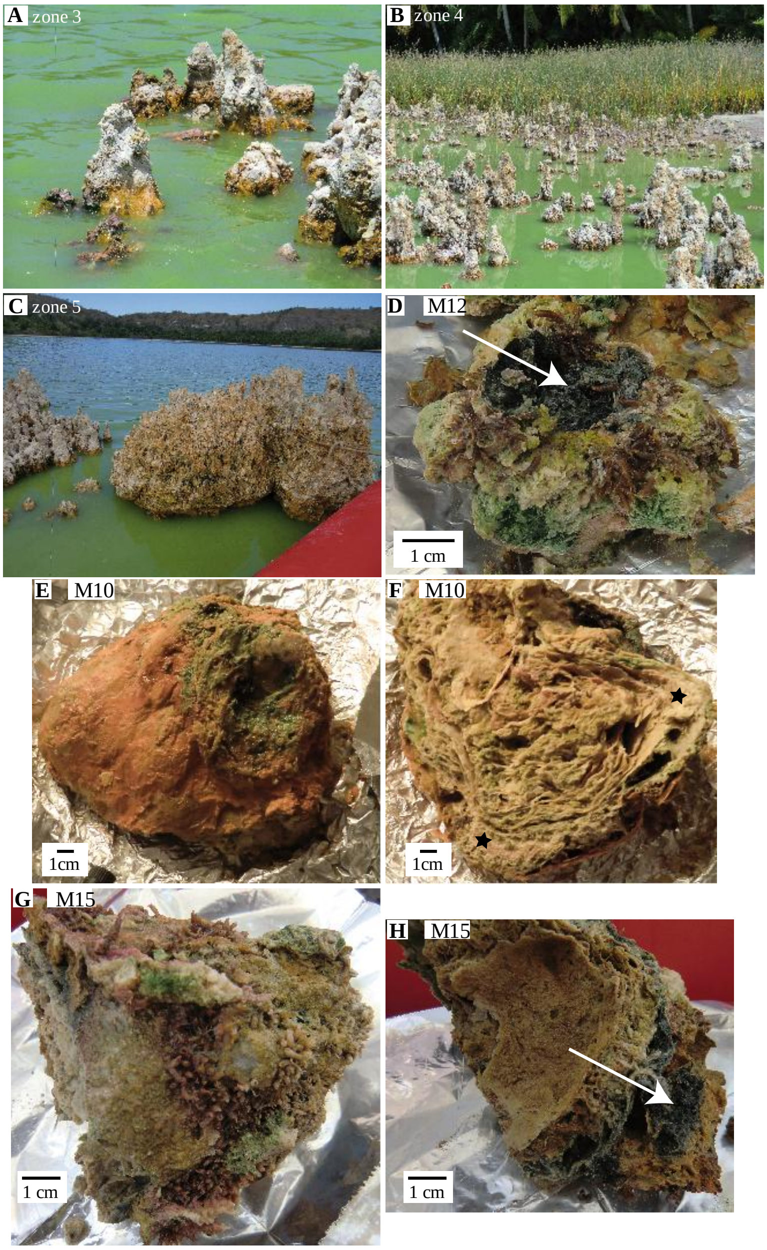

Figure S3: pictures of columnar stromatolites near the west and the south shores. At the time of collection, gas bubbling was detected next to M10 and M15. Different surface textures were observable, from totally smooth (M10 panel E) to more granular (M12 panel D and M15 panel G). Millimetric laminations were detected in all stromatolites (panels H an d F). The microbial mats associated with M12 and M15 have similar coloration with green (Cyanobacteria) and purple (purple bacteria) patches (panels D and G) and patches of fly pupae were abundant. Black areas were also visible inside those two stromatolites (arrows in panels D and H). The M10 stromatolite was texturally different than M12 and M15 and was covered by an orange microbial mat (panel E). Green and pink (black stars) laminations were alternating inside the M10 stromatolite (panel F). Recesses are observable in the center of those stromatolites (panels F and H) attesting that dissolution events have probably occurred internally.

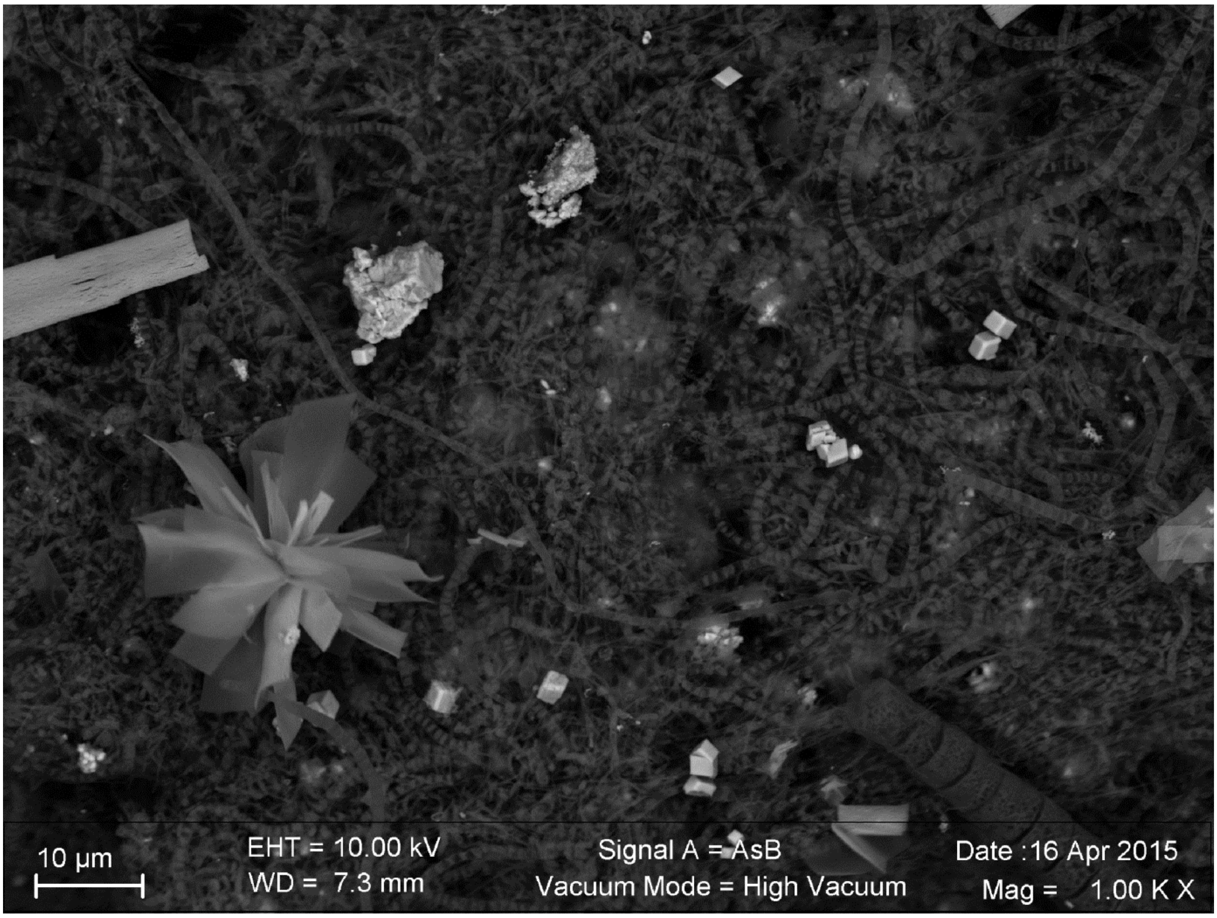

Figure S4: SEM image (at 10 KV, AsB detector) of the critical-point-dried microbial mat of M17 where filamentous cells are observed at the surface of the Pleurocapsales colonies. Empty areas were visible inside the filamentous cells probably corresponding to poly-β-hydroxybutyrate deposits. Calcium carbonates (bright) and rod of magnesium phosphate were associated with the mat and an *Arthrospira* filament is visible on the bottom right.

A

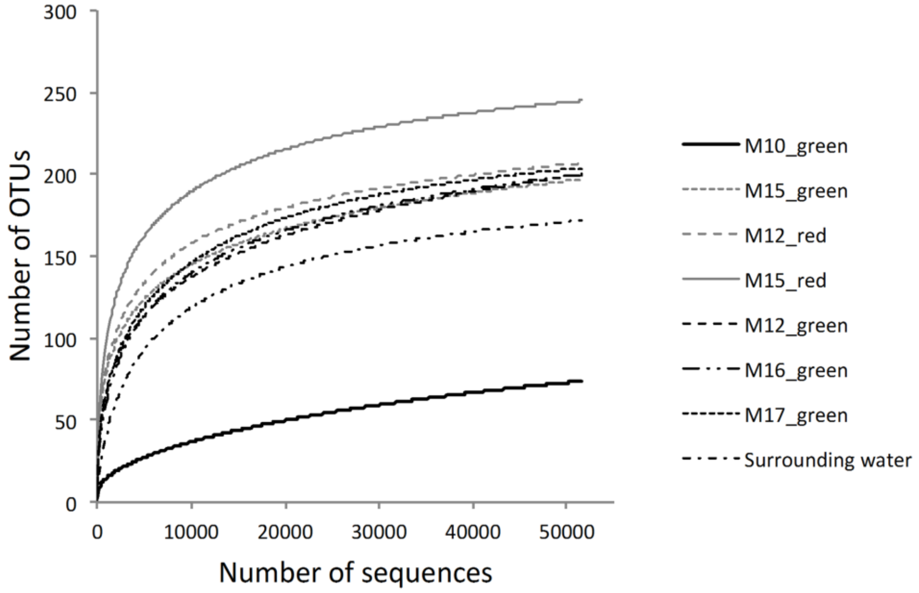

B

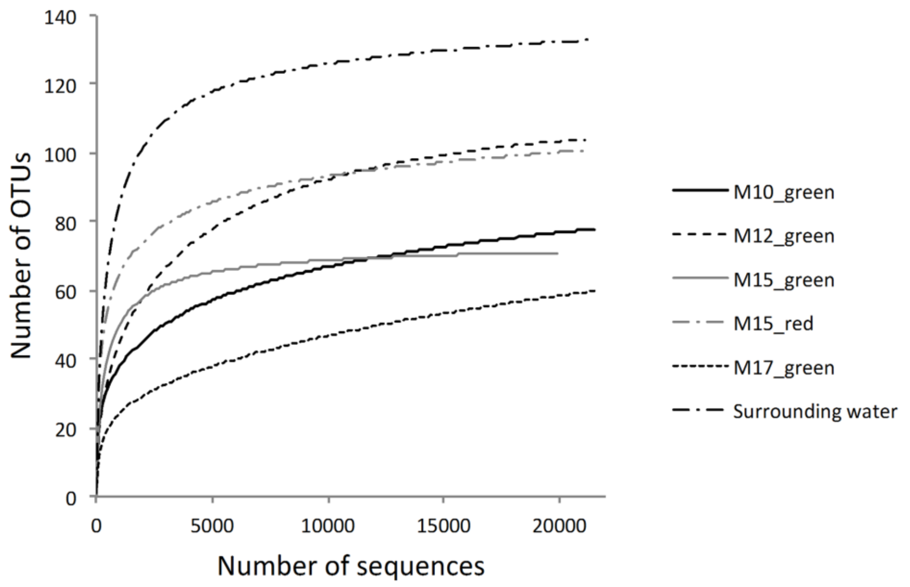

Figure S5: Rarefaction curves for Bacteria (A) and Archaea (B). Normalized datasets consisted in 51,730 sequences for Bacteria and 21,673 for Archaea

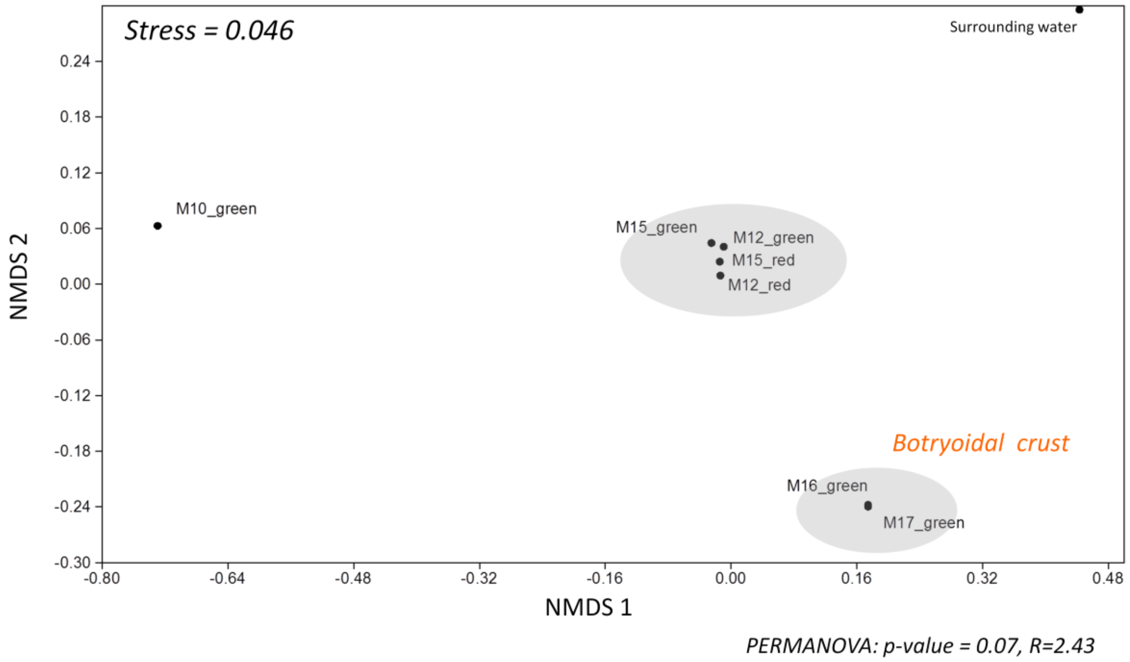

Figure S6: Non-metric multidimensional scaling (NMDS) ordination of bacterial community structure inferred from 16S rRNA gene counts from each OTU retrieved in Dziani Dzaha stromatolites.

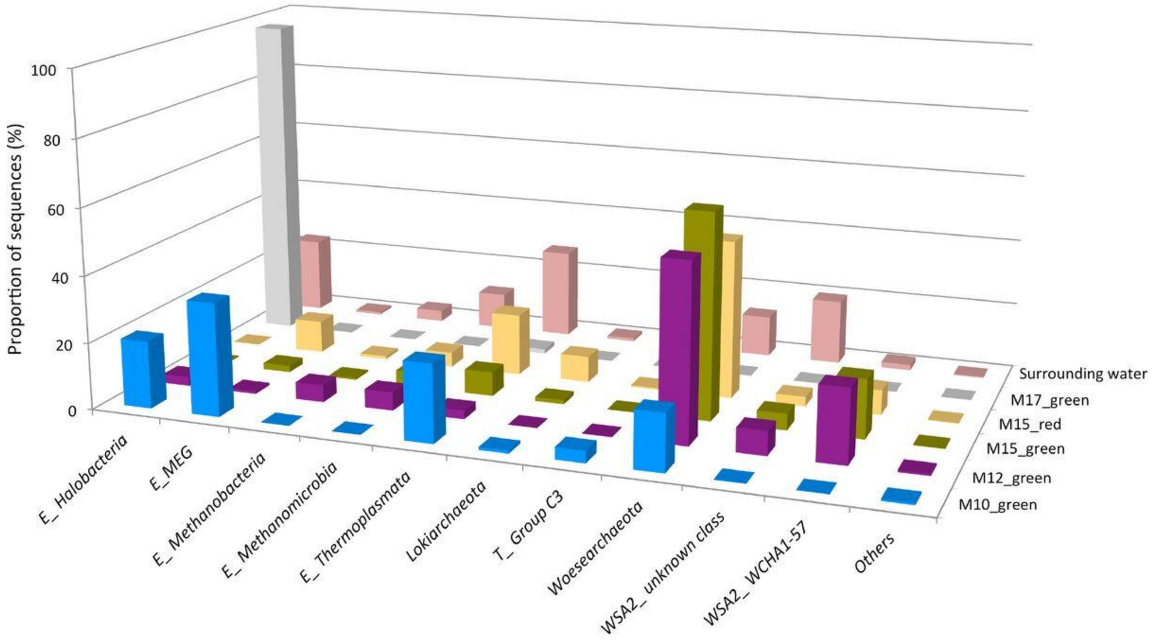

Figure S7: Proportion of sequences affiliated with major archaeal phyla (defined as > 0.2% of total sequence number).

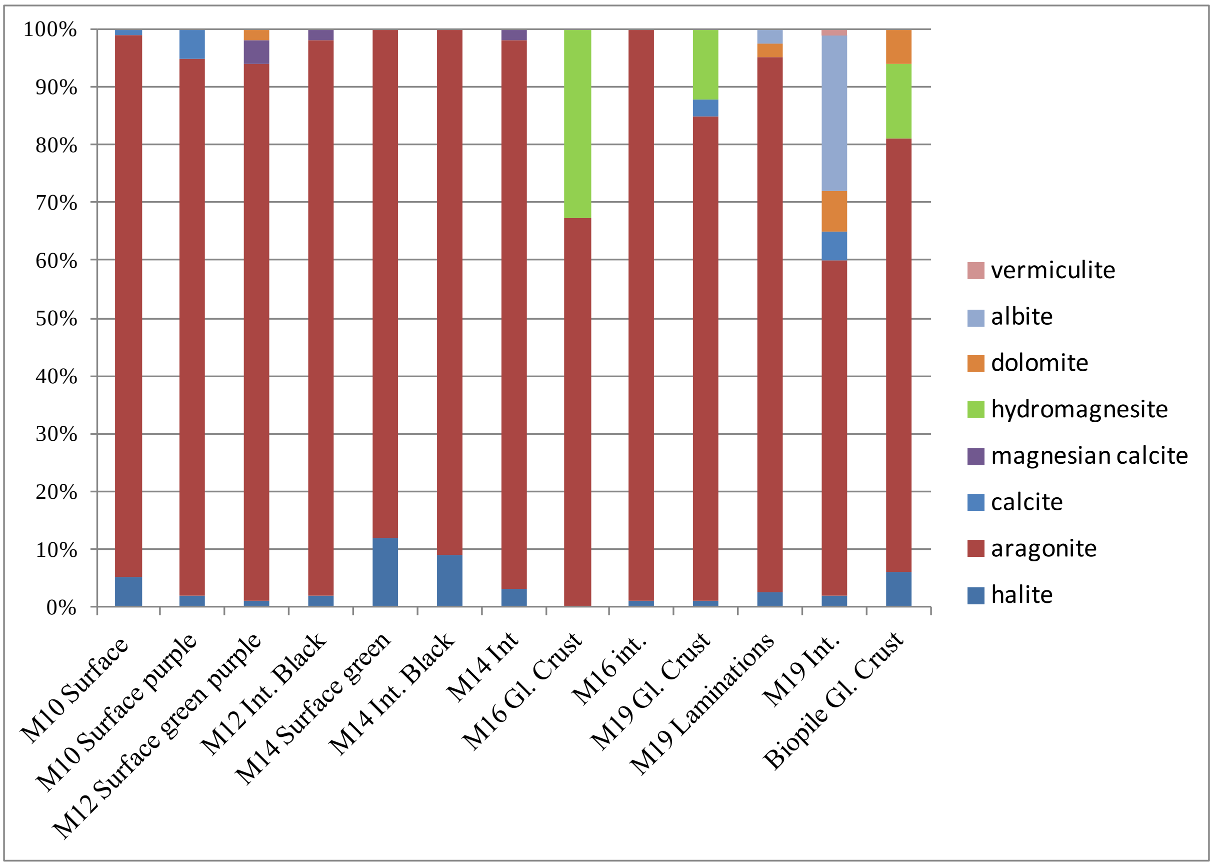

Figure S8: Proportion of the different mineral phases detected by X-ray diffraction analyze of the stromatolites from Dziani Dzaha Lake. Hydromagnesite was only detected in the globular crusts (Gl. crust). Int: internal part of the stromatolites. M14 has the same location and morphology as M15 collected for biological analyses. M19 was totally emerged at the time of collection and was a flat stromatolite with a cauliflower crust and a similar morphology to M17 collected next to it but under the water.

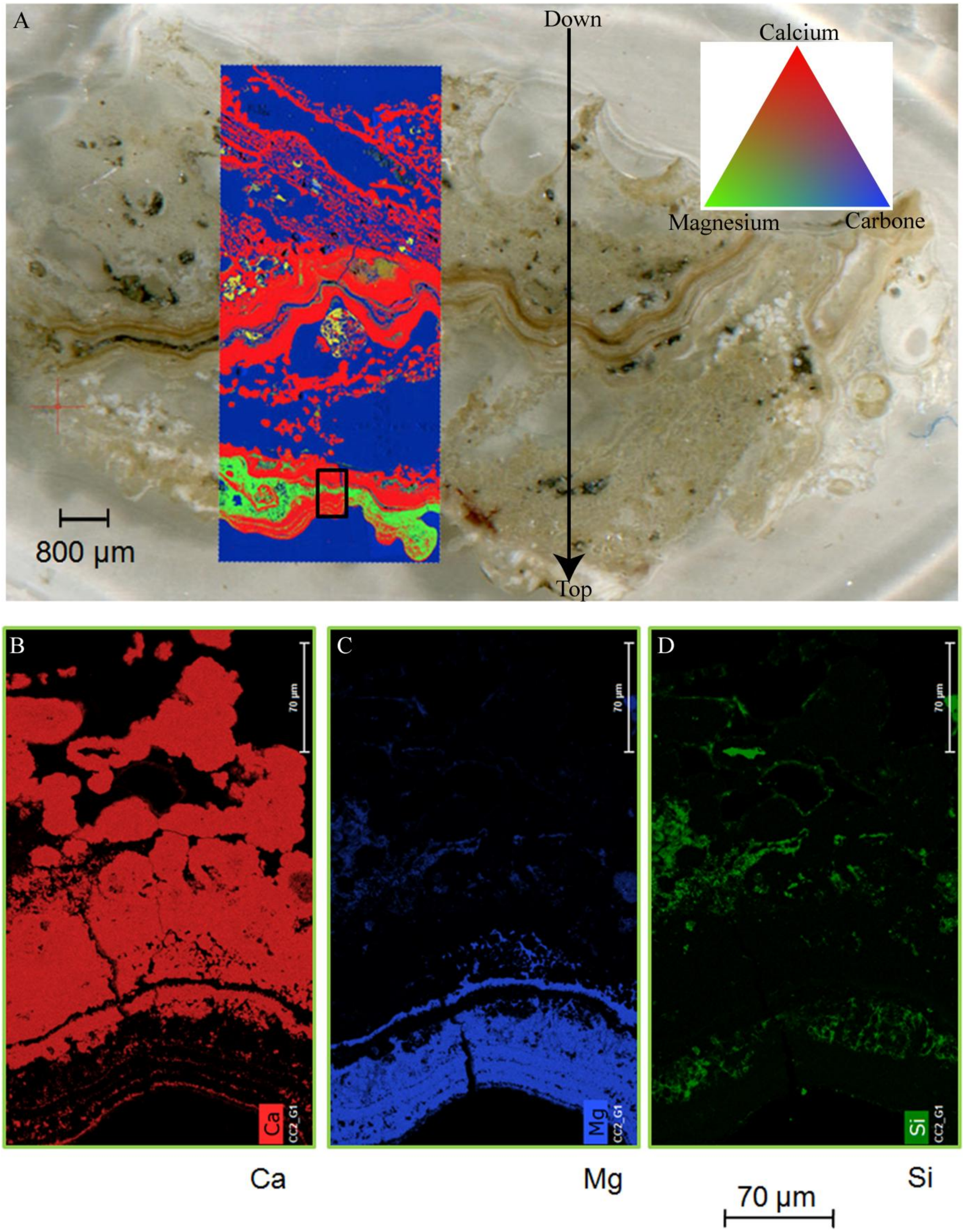

Figure S9: SEM-EDS map of the cauliflower-like crust and internal part of M17 transversal cutting embedded in Epoxy resin

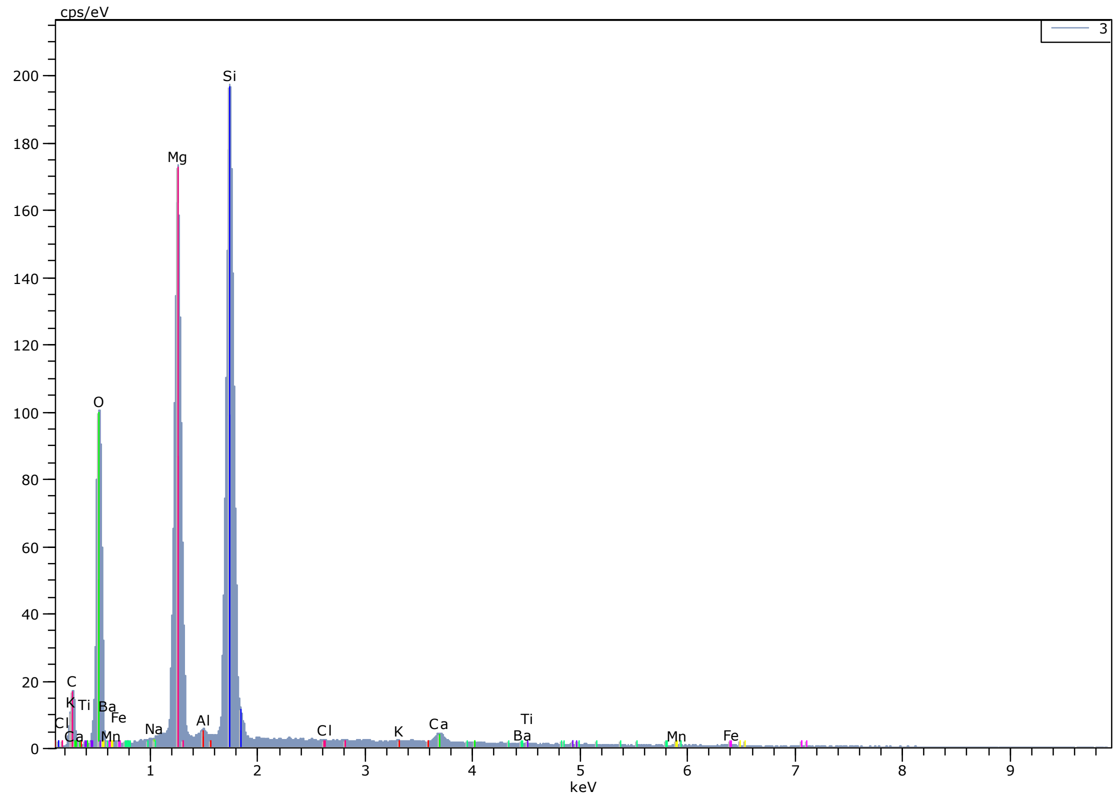

Figure S10: EDS spectrum of the magnesium silicate phase embedding Pleurocapsales in M17

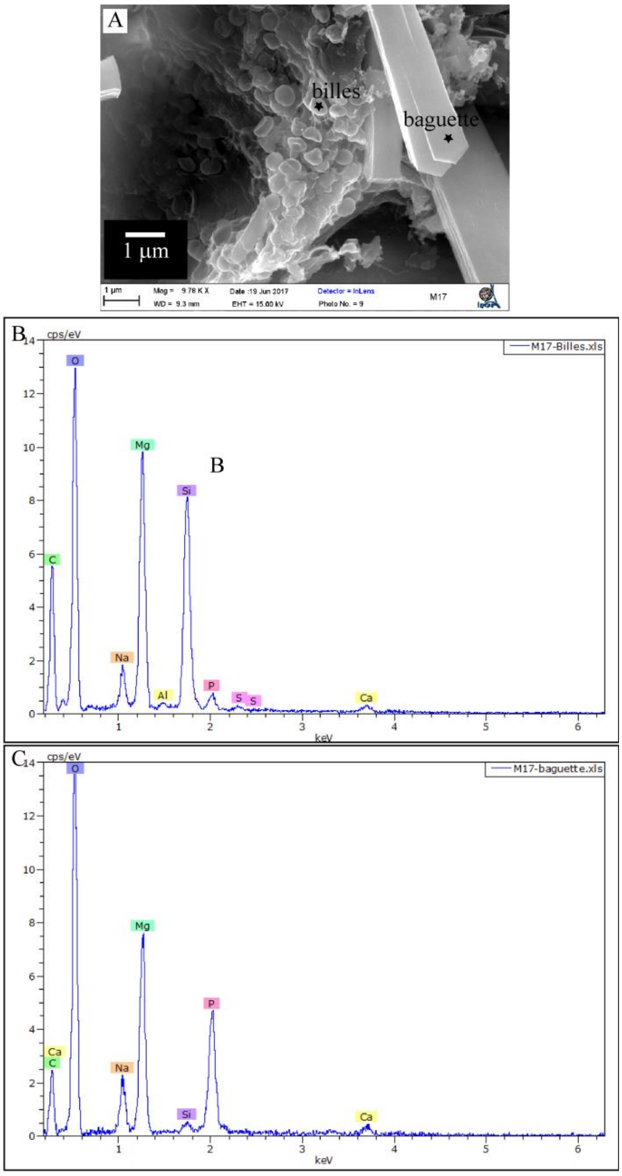

Figure S11: A) Secondary electron image of the Mg-Si beads (billes) and magnesium phosphate rods (baguette) associated with the micobial mat of M17 dessicated at he critical point. B) EDS spectrum of a magnesium silicate beads. C) EDS spectrum of one magnesium phosphate rod.

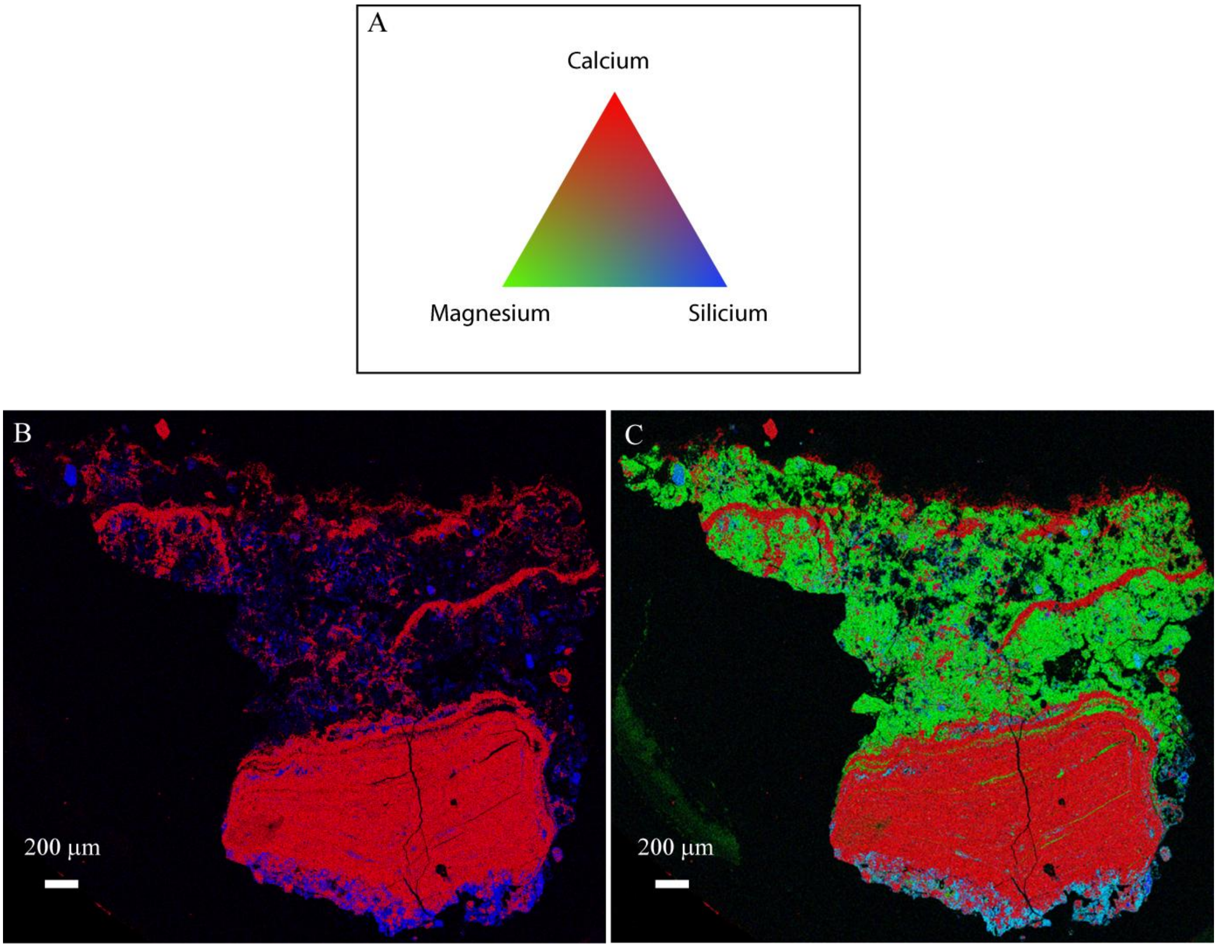

Figure S12: SEM-EDS map on M17 globular crust embedded in LR white resin showing silicon accumulation sometimes associated with an accumulation of magnesium in the sheath of Pleurocapsales. A) Colors associated with the SEM-EDS mappings. B) SEM-EDS mappings of calcium and silicon. C) SEM-EDS mappings of calcium, silicon and magnesium

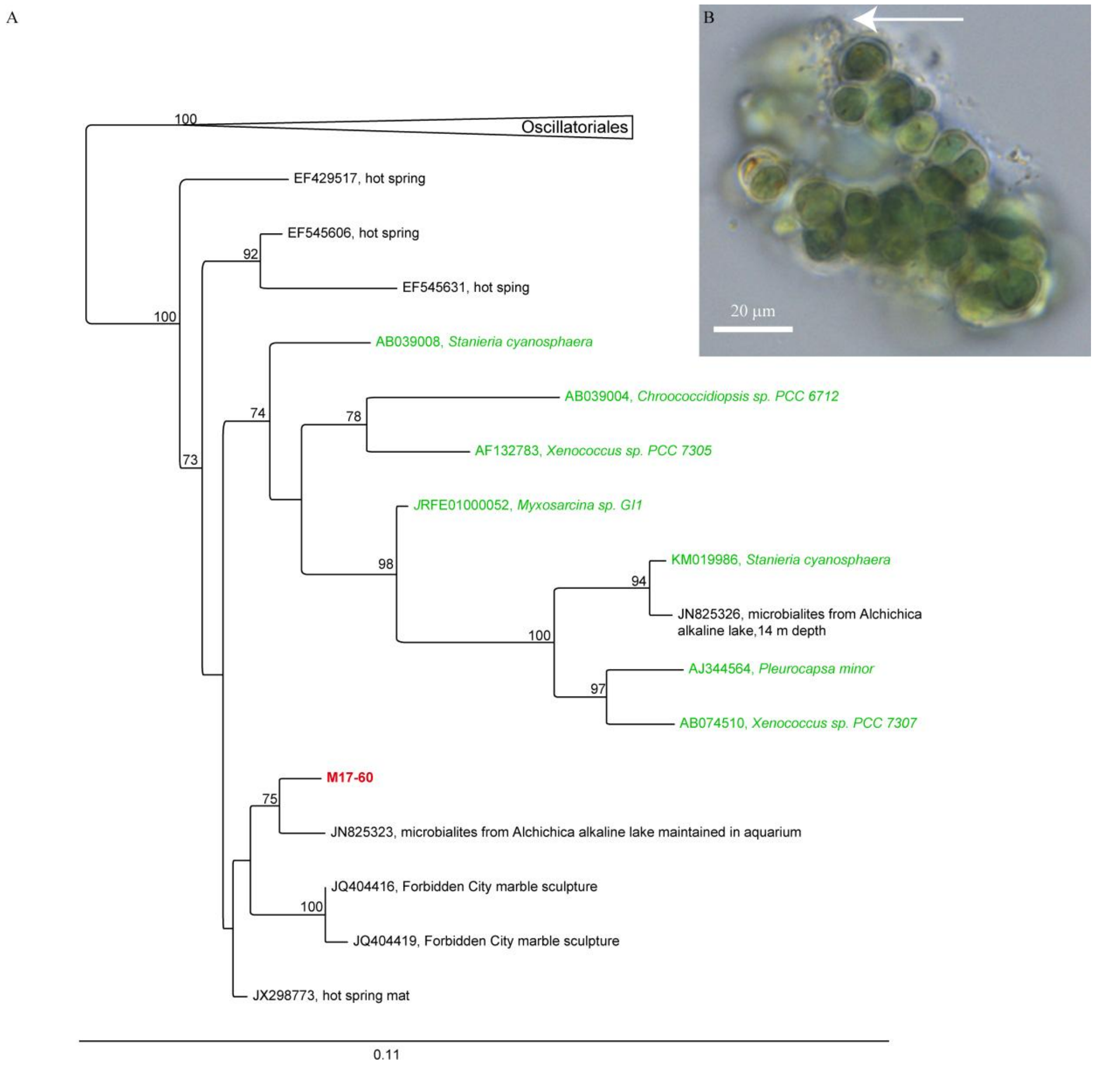

Figure S13: A) Phylogenic tree of the 16S RNA gene of the Pleurocapsales (M17-60) identified in consortia isolated by laser microdissection. B) Optical microscopy image of one of the consortia fixed with ethanol after laser microdissection showing the Pleurocapsales cells (green) surrounded by small coccoid cells (white arrow). The tree was constructed by maximum likelihood analysis, using 1,097 positions, including the closest possible uncultivated (black) and cultivated (green) relatives as well as more distant representatives of cultivated species. Bootstrap values for nodes (>70% support) based on 1,000 replicates are displayed as percentages. Nine Oscillatoriales 16S rRNA gene sequences were used for outgroup rooting.

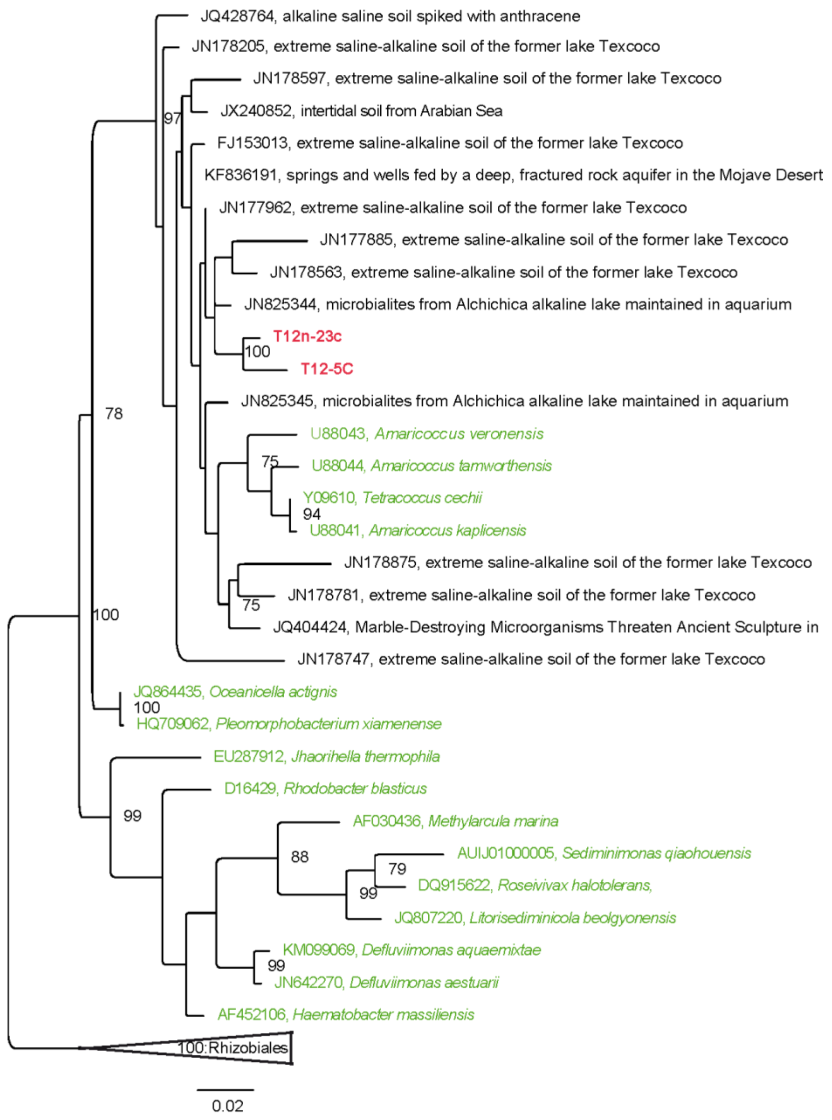

Figure S14: Phylogenic tree of the 16S RNA genes of the Rhodobacterales closely associated with the Pleurocapsales (T12n-23c and T12-5c) identified in two consortia isolated by laser microdissection. The tree was constructed by maximum likelihood analysis, using 1,116 positions, including the closest possible uncultivated (black) and cultivated (green) relatives as well as more distant representatives of cultivated species. Bootstrap values for nodes (>70% support) based on 1,000 replicates are displayed as percentages. 31 Rhizobiales 16S rRNA gene sequences were used for outgroup rooting
